# Digitizable therapeutics for decentralized mitigation of global pandemics

**DOI:** 10.1038/s41598-019-50553-x

**Published:** 2019-10-04

**Authors:** Adar Hacohen, Reuven Cohen, Sol Efroni, Baruch Barzel, Ido Bachelet

**Affiliations:** 1 Augmanity, Rehovot, Israel; 2 Faculty of Life Sciences, Bar-Ilan University, Ramat-Gan, Israel; 3 Department of Mathematics, Bar-Ilan University, Ramat-Gan, Israel

**Keywords:** Complex networks, Statistical physics

## Abstract

When confronted with a globally spreading epidemic, we seek efficient strategies for drug dissemination, creating a competition between supply and demand at a global scale. Propagating along similar networks, *e.g*., air-transportation, the spreading dynamics of the supply vs. the demand are, however, fundamentally different, with the pathogens driven by contagion dynamics, and the drugs by commodity flow. We show that these different dynamics lead to intrinsically distinct spreading patterns: while viruses spread homogeneously across all destinations, creating a concurrent global demand, commodity flow unavoidably leads to a highly uneven spread, in which selected nodes are rapidly supplied, while the majority remains deprived. Consequently, even under ideal conditions of extreme production and shipping capacities, due to the inherent heterogeneity of network-based commodity flow, efficient mitigation becomes practically unattainable, as homogeneous demand is met by highly heterogeneous supply. Therefore, we propose here a decentralized mitigation strategy, based on local production and dissemination of therapeutics, that, in effect, bypasses the existing distribution networks. Such decentralization is enabled thanks to the recent development of *digitizable* therapeutics, based on, *e.g*., short DNA sequences or printable chemical compounds, that can be distributed as digital sequence files and synthesized on location via DNA/3D printing technology. We test our decentralized mitigation under extremely challenging conditions, such as suppressed local production rates or low therapeutic efficacy, and find that thanks to its homogeneous nature, it consistently outperforms the centralized alternative, saving many more lives with significantly less resources.

## Introduction

In recent pandemics, from SARS to the West-African Ebola, we have fortunately averted a major global spread. However, when such scenraio will transpire, we will be challenged by a competition between the infectious pathogen and the therapeutic technology, each racing to reach the majority of the population first. This competition confronts us with several challenges: (i) the inevitable response time *t*_*R*_ required for us to instigate a mitigation plan places the pathogen at a potentially significant spreading advantage; (ii) while the pathogen reproduces as it spreads^1–3^, a therapy must be manufactured and shipped from one or few sources, whose production capacity may be limited^4–12^; (iii) under global demand we must ship the therapeutics worldwide, stretching the bounds of our limited transportation resources.

To assess our ability to address such a challenge we analyze the simultaneous dynamics of the two spreading processes: that of the pathogens versus that of the therapeutics. It would naïvely seem that winning this competition relies on rapid production and shipping capacity. However, here we show that due to the different spreading dynamics - drug dissemination becomes intrinsically inefficient when faced with global demand. The source of this inefficiency is rooted, not in production/shipping rates, but rather in the fact that network-based commodity flow leads to an uneven, and hence highly ineffective, supply of the therapeutic. As a consequence, we show that for a sufficiently large network, even unrealistically optimistic production/shipping capacities remain insufficient.

It seems, therefore, that the only viable strategy is to severely intervene in international mobility. On the one hand quarantining airports and restricting travel to halt the viral spread^13–15^, and on the other hand reshaping the dissemination network to allow a more even distribution of the therapeutic agent. Such major interventions, however, are not just impractical, but may also lead to significant economic loss and major political stress – indeed, *a lesser of two evils*, but still a potentially hurtful toll on global stability.

To break this gridlock, we consider an alternative mitigation strategy, based on *decentralized production*, in which the therapeutics are locally synthesized at each destination. This bypasses the distribution *networks*, and allows a more leveled spread of the therapeutic. We consider the fact that such decentralization might, under some conditions, exhibit lower production capacity, if, for instance, the local synthesis is inefficient, or the therapeutic efficacy is sub-optimal. However, as we show that equality precedes capacity, we find that this strategy consistently prevails thanks to its egalitarian nature, even if inferior in many other relevant parameters, *e.g*., production rate, response time or therapeutic efficacy. *Hence, the merits of decentralization overcome its potential practical shortcomings, calling on us to urgently develop decentralized mitigation capabilities*.

Given the complexity of drug production, decentralization seems, at first glance, unfeasible – how can each local population manufacture their own therapeutics? However, recent breakthroughs suggest that decentralized mitigation is limited by perception, rather than by technology. Indeed, certain types of therapeutics can be converted into digital information, handled and distributed as data, and then locally *printed*, *i.e*. synthesized, at their designated destination (Box 1, Section A). Such digital shipping of, *e.g*., DNA sequences, vaccines or therapeutic agents, is, already in use^16–18^, hence the relevant technology is, in fact, currently available. However, at present, this technology remains unscalable under global demand. We, therefore, continue to lack decentralized mitigation capabilities, a lacuna that is primarily driven by the current absence of motivation to scale-up our printing capacity, as indeed, the crucial advantages of decentralization are yet unrecognized. Following our analysis below, exposing the unequivocal merits of decentralized mitigation, we wish to prompt its development as a future response to global epidemics. This entails advancing the already existing technological pathways towards practical implementation at a global scale – a goal that, given the appropriate motivation, we believe is within our reach. In Section A we present specific guidelines for economically viable decentralized mitigation, showing that, under achievable reduction in costs, an annual investment of 50–500 USD per individual is sufficient to set up the required infrastructure within approximately one decade.

## Results

**Figure 1:**
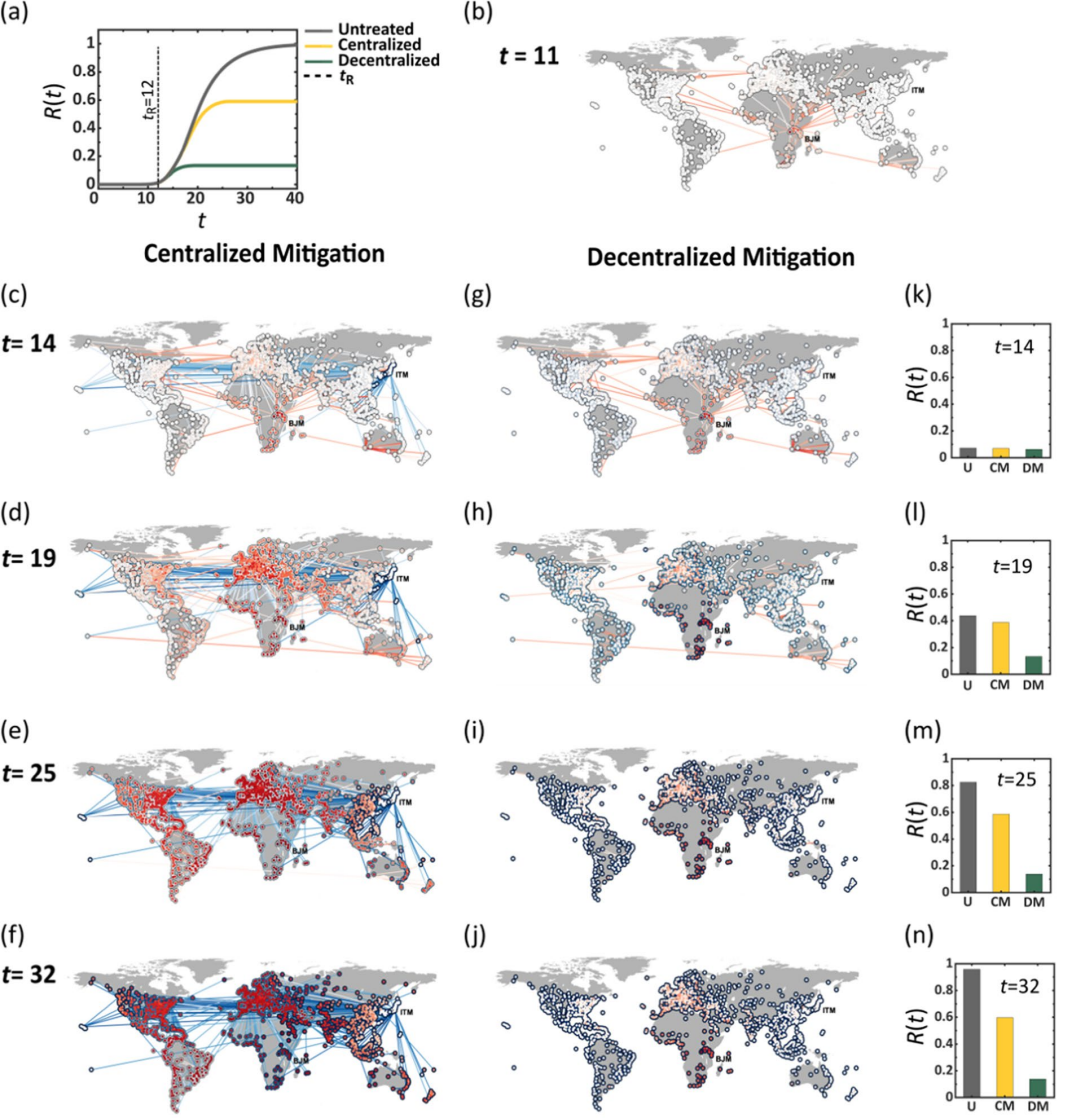
Outrunning a contageuous epidemic using Centralized vs. Decentralized therapeutic distribution. (**a**) The global coverage *R*(*t*) vs. *t* following an outbreak at Burundi (BJM). Lacking treatment we observe $R(t\to \infty )\sim 1$ (grey). Centralized mitigation, initiated at ${t}_{{\rm{R}}}=12$ days (dashed line), reduces mortality to ∼0.6 (yellow). Under identical conditions, decentralized mitigation achieves a four-fold increase in efficiency, with $R(t\to \infty )\sim 0.15$ (green). (**b**) The state of the epidemic at $t=11$, directly before drug dissemination begins. The local mortality levels ${r}_{n}^{{\rm{U}}}(t)$ in each node and the flux of infected individuals along each link (air-route) are represented by their red color depth. (**c**–**f**) Centralized drug dissemination begins at ${t}_{{\rm{R}}}=12$ in Osaka (ITM), with therapeutic fluxes (links) and drug availability levels (nodes circumference) represented by blue color depth. We observe a *race* between the therapeutic and the disease, both spreading along similar routes, ending in a significant fraction of infected individuals, as indicated by the many red nodes at *t* = 32. (**g**–**j**) In decentralized mitigation each node synthesizes its own pool of therapeutics (blue circumference), resulting in a dramatic reduction in mortality, with the only *deep red* nodes, being the ones in the vicinity of BJM, that were impacted prior to our response at ${t}_{{\rm{R}}}=12$. Here we observe no therapeutic flux along the links, as the therapeutic is disseminated digitally, bypassing the physical transportation network. (**k**–**n**) *R*(*t*) at the four selected time points under no treatment (U, grey), with centralized mitigation (CM, yellow) and under decentralized mitigation (DM, green). Here and throughout we set the parameters in Eq. (12) to $\alpha =2$ day^−1^, $\beta =0.2$ day^−1^, $\gamma =1.0$, $\zeta =1.0$ day^−1^, $h=8$ and $\varepsilon ={10}^{-6}$. The mean capacities under both centralized (15) and decentralized (16) mitigation were set to $\langle {c}_{n}\rangle ={C}_{s}=0.2$ day^−1^ and the response time to $\,{t}_{R}=12$ days. The individual capacities *c*_*n*_ are extracted from a normal distribution ${\mathscr{N}}(\mu ,{\sigma }^{2})$ with mean $\mu =0.2$ and standard deviation $\sigma =0.1\,\mu$.

To demonstrate the potential utility of our proposed decentralized response we consider different epidemiological scenarios, from mildly contagious to extremely virulent, in which a lethal (or otherwise irreversible) epidemic spreads globally via air-travel, under the susceptible-infected-removed (SIR) epidemic model^19–21^ (Box 2). We used empirical data on human aviation to evaluate the flux of passengers between $N=1,292$ local populations (nodes), each with *M*_*n*_ individuals ($n=1,\ldots ,N$), and quantified the impact of the epidemic through its global *coverage*

1 $$R(t)=\frac{{\sum }_{n=1}^{N}{M}_{n}{r}_{n}^{{\rm{U}}}(t)}{{\sum }_{n=1}^{N}{M}_{n}},$$

where ${r}_{n}^{{\rm{U}}}(t)$ is the fraction of *removed untreated* individuals in *n*. For an extremely contagious disease, absent any treatment, we have $R\equiv R(t\to \infty )\to 1$, representing the infection of the entire population (Fig. 1a, grey). Such extreme scenario, while unlikely, helps us challenge our examined mitigation strategies, putting them to the test under most adversarial conditions. Other, less virulent, scenarios, including epidemiological parameters extracted from common diseases, such as the flu, are analyzed in Supplementary Section 4.2, leading to similar findings.

Following the initial outbreak at *t* = 0, we define the response time *t*_R_ as the time required to begin the distribution of a therapy. We simulated two different therapeutic scenarios, both beginning at $t={t}_{{\rm{R}}}$:

### Centralized mitigation

We take the classic approach, in which the developed drug is manufactured at a specific source node *s*, then distributed globally via air-transportation (Fig. 1c–f). In each location, some of the drug is consumed, based on the local infection levels, and the rest continues to travel to farther destinations, through pre-planned travel paths from *s* to all other nodes (Supplementary Section 2.2). In this approach, the dissemination is limited by the source’s distribution capacity *C*_*s*_ (day^−1^), capturing the number of doses that can be shipped from *s* per day, as dictated both by *s*’s manufacturing capabilities and by the carrying capacity of the global transportation network. Setting ${C}_{s}=1$ represents a scenario where *s* is capable of distributing sufficient supply to satisfy the global demand in a single day, *i.e*. produce and ship doses at a volume comparable to the entire global population. Most commonly we expect to have ${C}_{s} < 1$.

In Fig. 1c–f we present the evolution of the epidemic at four selected time-points. At *t* = 0 we simulate an outbreak (red) at Burundi (BJM), emulating the 2013 Ebola, which originated in Africa^22,23^, then track its spread through air-travel. The node infection levels and the epidemic fluxes, *i.e*. the daily volume of infected passengers on each route, are represented by red color depth. Drug dissemination (blue) begins at ${t}_{{\rm{R}}}=12$ days in Osaka (ITM), again using blue color depth to signify the availability/flux of drugs in each node/route. The snapshots illustrate the competition between the two spreading processes – the diffusing pathogen vs. the disseminated therapeutic – showing, through the long-term prevalence of infections (red) the inefficiency of centralized mitigation in the face of the globally spreading epidemic.

### Decentralized mitigation

In the decentralized scenario, the digital therapeutic is sent out as data, reaching all destinations practically instantaneously at $t={t}_{{\rm{R}}}$ (Fig. 1g–j). Here, the main *bottleneck* for mitigation is driven by the local rates *c*_*n*_ ($n=1,\ldots ,N$), capturing each node’s capacity to synthesize and locally disseminate the digital sequence in its material form. The capacity *c*_*n*_ is impacted by the abundance of printing devices in *n* and by the logistic efficiency of *n*’s local health-care system in delivering the printed drugs to the infected population. Hence ${c}_{n}=1$ translates to a daily production and dissemination of *M*_*n*_ doses per day, *i.e*. covering the entire local demand.

For comparison purposes, note that a *mean capacity* of $\langle {c}_{n}\rangle =C$ captures a state in which the decentralized production covers, overall, a *C*-fraction of the global demand per day, equivalent, under centralized mitigation to setting ${C}_{s}=C$. Therefore, $\langle {c}_{n}\rangle ={C}_{s}=C$ represents a scenario where both strategies, centralized vs. decentralized, exhibit similar global production rates, isolating only the effect of the decentralization.

The results of the decentralized strategy are shown in Fig. 1g–j. As before, the spread of the disease is captured by the red nodes and links, however, in this case, the drug no longer progresses along the *network*, but rather manufactured locally at rates *c*_*n*_, therefore, the blue links are absent. Instead, drug availability in each location is signified by the blue color depth of each node’s circumference, while infection levels are, as above, captured by the red fill of all nodes. Using similar lag *t*_R_ and capacities $\langle {c}_{n}\rangle ={C}_{s}=0.2$, *i.e*. a daily coverage of 20%, we find that decentralization is by far more efficient. In this example, the total infected population is reduced from ${R}_{0}\approx 1$ under no treatment, to $R=0.15$ under decentralized mitigation. This is compared to $R=0.60$, four times higher, under the traditional centralized strategy (Fig. 1a,k–n).

### Quantifying mitigation efficiency

To systematically asses the performance of both strategies, we track the mitigation efficiency via

2 $${\epsilon }=1-\frac{R}{{R}_{0}},$$

where *R*, taken from (1), is the observed long term coverage under centralized/decentralized treatment and *R*_0_ is the projected coverage in the absence of treatment, *i.e*. ${C}_{s}=\langle {c}_{n}\rangle =0$. A perfect response is captured by ${\epsilon }\to 1$, *i.e*. $R\ll {R}_{0}$, representing a dramatic reduction in the disease coverage. Conversely, ${\epsilon }\to 0$ indicates that infection levels remained almost unchanged by our intervention.

**Figure 2:**
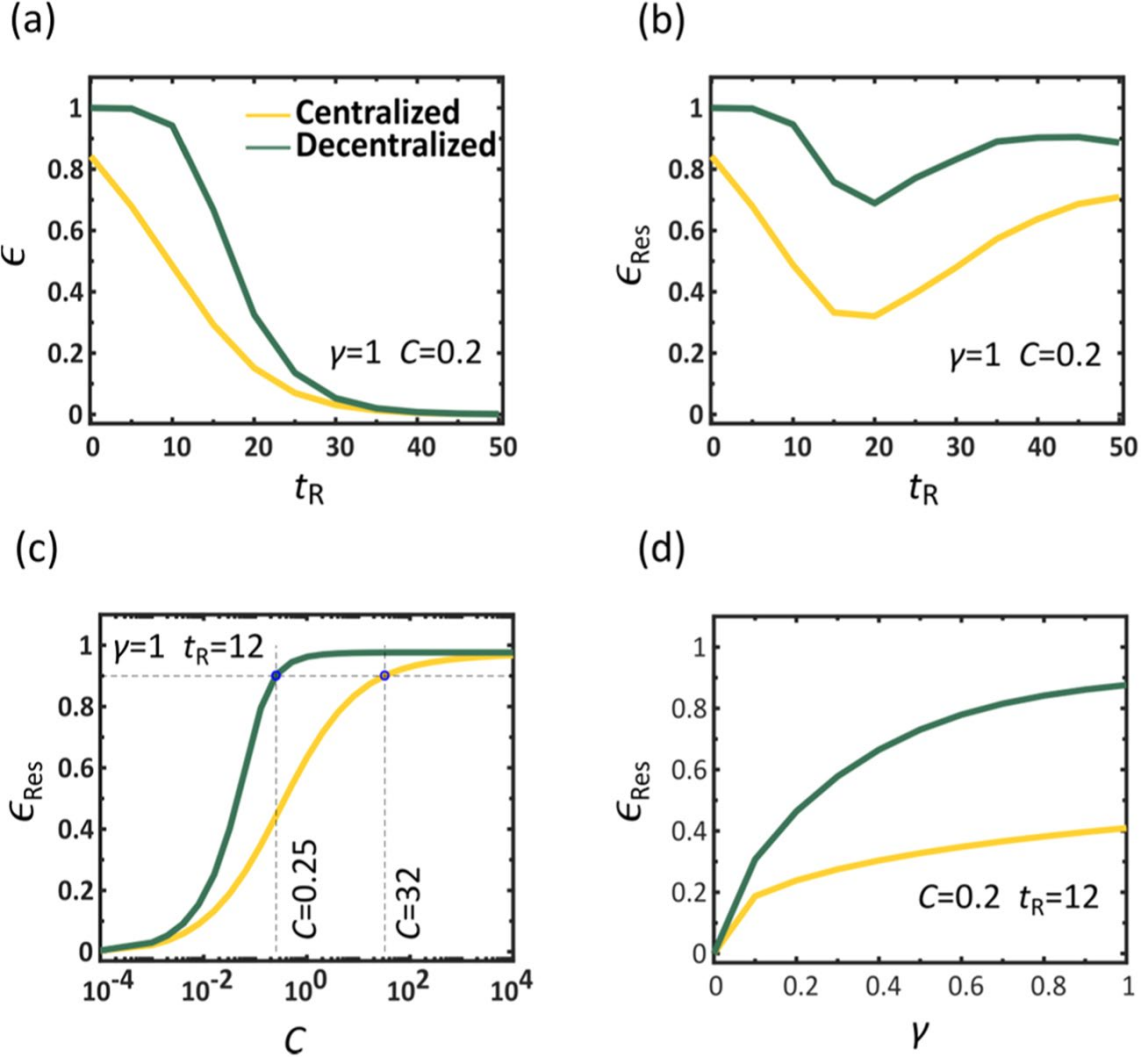
Efficiency of Centralized vs. Decentralized mitigation. The efficiency ${\epsilon }$ vs. the response time *t*_R_ under centralized (yellow) and decentralized (green) drug dissemination, showing that centralization is consistently superior. For large *t*_R_ both methods show little efficiency as the majority of the population has already been impacted. (**b**) The residual efficiency ${{\epsilon }}_{{\rm{Res}}}$ (3) vs. *t*_R_, capturing the reduction in mortality posterior to our response, namely eliminating individuals that have already perished prior to *t*_R_. We find that even under late response (large *t*_R_), decentralized (green) still saves a larger fraction of the *remaining* population compared to centralized (yellow). (**c**) ${{\epsilon }}_{{\rm{Res}}}$ vs. the capacity $\langle {c}_{n}\rangle ={C}_{s}=C$, which captures the fraction of the global demand that can be manufactured and disseminated daily. We find that a successful centralized mitigation requires unrealistic production and shipping capacities (yellow). For instance, to achieve ${{\epsilon }}_{{\rm{Res}}}=0.9$, a 90% reduction in post-response mortality, we must have a capacity of $C > 30$, *i.e*. distribute doses in excess of 30 times the global demand per day (dashed lines). Strikingly, a similar efficiency can be achieved under decentralized mitigation (green) with capacity as low as $C=0.25$, representing a two orders of magnitude reduction in capacity, while achieving a comparable outcome. Therefore, decentralization is not only faster, using digital data transmission instead of physical shipping routes, but also inherently more efficient, saving more people with significantly less, and hence realistic, resources. (**d**) ${{\epsilon }}_{{\rm{Res}}}$ vs. drug efficacy *γ*. The locally synthesized drugs (green) achieve higher performance compared to the centrally distributed ones (yellow) even if their therapeutic efficacy is low. In fact, as long as the digital therapeutic has $\gamma  > 0.2$, namely that only one out of five individuals is cured by the drug, it is guaranteed to exceed the performance of the centralized treatment, even if the latter has a 100% success rate ($\gamma =1$). Here in each panel we varied a specific parameter (${t}_{{\rm{R}}},\,C$ or *γ*) while controlling for all others, as appears in the panels themselves and detailed in the caption of Fig. 1.

A crucial factor impacting our mitigation outcome, is the response time *t*_R_, required to identify the threat and initiate a response. To observe this, in Fig. 2a we present ${\epsilon }$ vs. *t*_R_ for both centralized (yellow) and decentralized (green) mitigation. As expected, we find that ${\epsilon }$ declines with *t*_R_, however, for the entire range of response times decentralization consistently achieves higher efficiency. In fact, even in the ideal case, where ${t}_{{\rm{R}}}=0$, an *immediate* response, centralized distribution achieves an efficiency of only 80%, while decentralization spares practically all potential infections.

In the limit of large *t*_R_ both methods exhibit low efficiency, a natural consequence of the fact that the majority of the impacted population has already perished, and can no longer be treated. Therefore, we consider the residual coverage $\Delta R=R-R({t}_{{\rm{R}}})$, capturing only the fatalities that occurred posterior to our response. This allows us to evaluate the residual efficiency via

3 $${{\epsilon }}_{{\rm{Res}}}=1-\frac{\Delta R}{\Delta {R}_{0}},$$

where $\Delta {R}_{0}={R}_{0}-{R}_{0}({t}_{{\rm{R}}})$. We now see that even if *t*_R_ is large, our ability to save the *remaining* population is enhanced if we prioritize decentralized over centralized mitigation (Fig. 2b).

Next, we examine the impact of *capacity* on the efficiency of the disease mitigation. We consider a spectrum of capacities ${C}_{s}=\langle {c}_{n}\rangle =C$, with *C* ranging from 10^−4^ to ${10}^{4}\,{{\rm{day}}}^{-1}$, spanning a broad range, from extreme deprivation to extreme overproduction. We find, again, that decentralization is significantly more efficient (Fig. 2c, green), achieving an efficiency of ${{\epsilon }}_{{\rm{Res}}} > 0.9$ already at $C=0.25$, a scenario in which the average node can only produce 25% of its demand per day (dashed-lines). Similar efficiency under centralized distribution (yellow) is only achieved at $C \sim 30$, which is not only 10^2^ times higher than the decentralized alternative, but, most importantly, an extremely unrealistic value, describing a state in which a single source node *s* produces and ships enough doses per day to cover 30 times the global demand. Optimal efficiency ${{\epsilon }}_{{\rm{Res}}}\to 1$, achieved around $C\sim 1$ with decentralization, is only reached under the completely unattainable $C\sim {10}^{3}$ in the case of centralized distribution. Another crucial factor we examine is the therapeutic efficacy *γ*, quantifying the probability of recovery after receiving the physical/digital treatment. Once again, we find that decentralization is superior, achieving a higher ${ {\mathcal E} }_{{\rm{Res}}}$, even with significantly lower efficacy *γ* (Fig. 2d).

Together, we find that decentralized mitigation, based on digitizable therapeutics and local synthesis, achieves a dramatically higher reduction in infection/mortality under significantly lower, and therefore realistic, production (*C*) or efficacy (*γ*) levels. Counter-intuitively, these results are unrelated to the faster dissemination of digital compared to physical media. Indeed, this distinction between the *speed* of data versus that of physical commodities was not even introduced into our modeling of the dissemination in Eqs. (15) and (16), and hence, it plays no role in the decentralized advantage. We, therefore, conclude that decentralization provides intrinsic merits that go beyond the classic measures of production rates or shipping capacity. Below, we explore these merits, showing that they are deeply rooted in the egalitarian nature of localized production, as opposed to the intrinsically unequal distribution of network-based commodity flow.

### Inequality and mitigation efficiency

**Figure 3:**
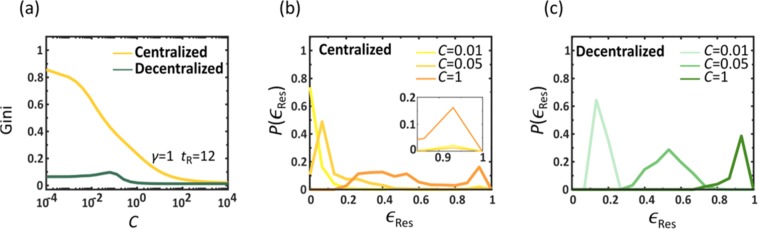
Equality and allocation of resources in drug dissemination. To understand the roots of the dramatically improved performance of decentralized vs. centralized we examined the level of inequality in the local efficiencies ${{\epsilon }}_{{\rm{Res}},n}$ via the Gini coefficient (5). (**a**) Gini vs. *C* for centralized (yellow) and decentralized (green) mitigation. While the latter is egalitarian by nature, having a low Gini coefficient, the former is extremely uneven, with few nodes that benefit from high ${{\epsilon }}_{{\rm{Res}},n}$ and a majority of nodes that remain deprived. This indicates that uneven allocation of resources (Gini), and not the slower production/shipping rates (*C*), is the main obstacle for efficient mitigation. (**b**) The probability density $P({{\epsilon }}_{{\rm{Res}}})$ under centralized mitigation, as obtained for different capacity levels *C*. We observe a coexistence of a minority of well treated nodes (peak around ${{\epsilon }}_{{\rm{Res}}}\approx 1$, see inset) and a majority of nodes with varying efficiency levels. (**c**) In contrast, under decentralized mitigation we observe a bounded $P({{\epsilon }}_{{\rm{Res}}})$, whose mean efficiency (peak) approaches ${{\epsilon }}_{{\rm{Res}}}=1$ uniformly as *C* is increased. This represents an even allocation of benefits, in which most nodes witness a similar rise in efficiency as capacity is increased.

To examine the impact of our response at each individual node, we measured the local residual efficiency

4 $${{\epsilon }}_{{\rm{Res}},n}=\,1-\frac{\Delta {r}_{n}^{{\rm{U}}}}{\Delta {r}_{n,0}},$$

where $\Delta {r}_{n}^{{\rm{U}}}={r}_{n}^{{\rm{U}}}(t\to \infty )-{r}_{n}^{{\rm{U}}}({t}_{{\rm{R}}})$ and $\Delta {r}_{n,0}={r}_{n,0}(t\to \infty )-{r}_{n,0}({t}_{{\rm{R}}})$ represent the residual mortality in *n* with and without the therapeutic, respectively. In analogy with Eq. (3), this local efficiency quantifies the *benefit* provided by the disseminated therapeutic to each specific location *n* on a scale ranging from zero (no benefit) to unity (optimal). This allows us to evaluate the benefit inequality across all nodes through the Gini coefficient^24,25^

5 $${\rm{Gini}}=\frac{1}{2N}\frac{{\sum }_{n,m=1}^{N}|{{\epsilon }}_{{\rm{Res}},n}-{{\epsilon }}_{{\rm{Res}},m}|}{{\sum }_{n=1}^{N}{{\epsilon }}_{{\rm{Res}},n}},$$

which ranges from zero, for a fully uniform ${{\epsilon }}_{{\rm{Res}},n}$, to unity, in the limit of extreme inequality. We find in Fig. 3a that under decentralized mitigation the inequality is small, with Gini being close to zero independently of *C* (green). In contrast, centralized mitigation (yellow) creates an inherent unevenness, exhibiting a high Gini coefficient even when $C\sim 1$. To get deeper insight we calculated the probability density $P({\epsilon })$ for a randomly selected node to have ${{\epsilon }}_{{\rm{Res}},n}\in ({\epsilon },{\epsilon }+{\rm{d}}{\epsilon })$. In centralized mitigation (Fig. 3b) we observe for $C=0.01$ (yellow) a high density around ${\epsilon }\to 0$, and a slight increase in $P({\epsilon })$ close to unity (see inset). This depicts a coexistence of a majority of low efficiency nodes with a selected minority of saved nodes, for which ${{\epsilon }}_{{\rm{Res}},n}$ is high, illustrating the severe benefit inequality. Only when *C* is set to 1 (orange) do we observe the highest density at ${{\epsilon }}_{{\rm{Res}},n}\approx 1$. Yet even under these conditions, the saved nodes continue to coexist alongside a long tail of underserved destinations whose local efficiency reaches as low as 0.2. In contrast, under decentralized mitigation, $P({\epsilon })$ exhibits a uniform shift towards ${\epsilon }=1$ as *C* is increased, representing an *egalitarian* decrease in mortality, evenly spread across all populations (Fig. 3c).

### Analytical results: the spreading advantage of viruses versus therapeutics

Centralized mitigation is, in its essence, a spreading competition between the therapeutics and the pathogens, both progressing along the same underlying network, *i.e*. air-transportation. It seems, naïvely, that winning this competition is a matter of propagation efficiency: we must manufacture and ship therapeutics at sufficient rates to outrun the spread of the disease. However, the phenomenological analysis above indicates, that there is an intrinsic deficiency in the spread of therapeutics, that cannot be easily compensated by simply increasing production/shipping rates *C*_*s*_. Indeed, as we next show, the two competing processes – viruses vs. therapeutics – lead to fundamentally different spreading patterns, in which the viruses benefit from an intrinsic advantage.

#### Viral spread

**Figure 4:**
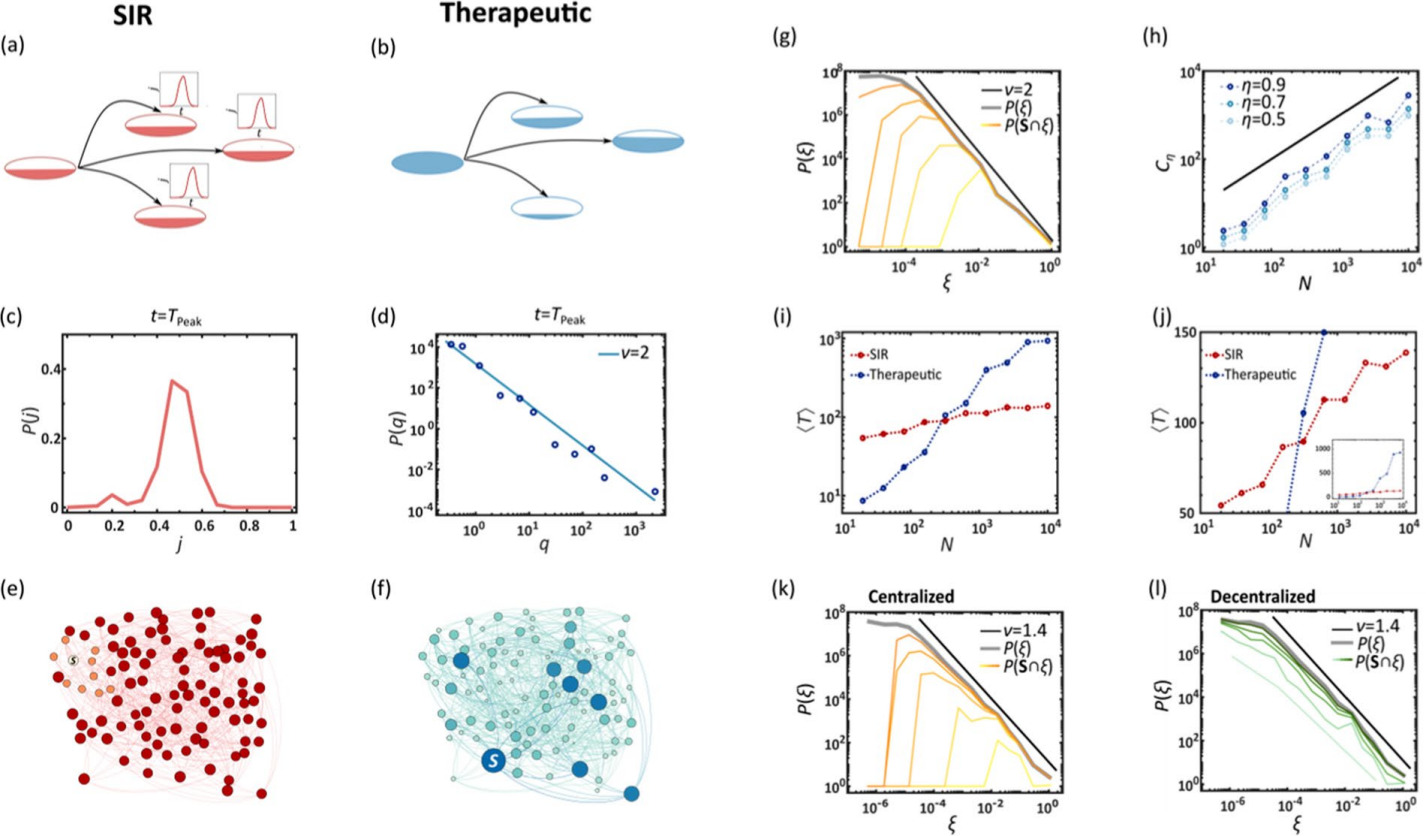
Spreading competition between pathogens and therapeutics. (**a**) As it spreads, the pathogen endogenously reproduces identically at each node through SIR. (**b**) In contrast, the therapeutic flows in different rates into each node. We analyze the resulting spreading patterns on a weighted random network of $N={10}^{4}$ nodes ($k=10$, weights extracted from ${\mathscr{N}}(1.0,{0.2}^{2})$). (**c**) The probability density *P*(*j*) for a randomly selected node to have infection level *j*, as obtained at $t={T}_{{\rm{Peak}}}$. The bounded nature of *P*(*j*) indicates that the global demand is homogeneous. (**d**) At the same time the supply of the therapeutic is extremely heterogeneous with $P(q)\sim {q}^{-2}$, depicting a coexistence of deprived and oversupplied nodes. (**e**,**f**) These distinct spreading patterns are clearly visible in a featured small network of *N* = 100 nodes: The pathogens (red) impact all nodes roughly simultaneously and homogeneously, while the drug supply (blue) is highly heterogeneous, reaching few nodes early, and leaving most nodes to lag behind. This disparity between homogeneous demand and heterogeneous supply – an intrinsic characteristic of the competing spreading processes – severely limits the effectiveness of centralized mitigation. (**g**) We measured the rates *ξ*_*sn*_ in (6) and obtained the probability density *P*(*ξ*) for ${\xi }_{sn}\in (\xi ,\xi +{\rm{d}}\xi )$ (grey). As predicted we find that $P(\xi )\sim {\xi }^{-2}$ (black), exposing the topological roots of the observed supply heterogeneity. Dividing the nodes into saved (${\bf{S}}=1$) and unsaved (${\bf{S}}=0$) we also measured $P({\bf{S}}\cap \xi )$ under varying capacity levels *C* (yellow to orange, $C=0.001,0.005,0.02,0.04,0.1$ days^−1^). As expected, the saved nodes tend towards the large *ξ* tail of the distribution, showing that, indeed, the broad distribution of *ξ*_*sn*_ determines the mitigation efficiency. (**h**) The critical capacity ${C}_{\eta }$ required to save (${\bf{S}}=1$) an $\eta$ fraction of all nodes vs. the number of nodes *N*. As predicted in Eq. (8) we observe ${C}_{\eta }\sim {N}^{\varphi }$, a scaling relationship that renders mitigation unattainable for large networks ($N\to \infty$). For a random network we predict $\varphi =1$ (solid black line). (**i**,**j**) The mean spreading time 〈*T*〉 of the pathogen (red) and the therapeutic (blue) vs. system size *N*. The therapeutic spreads in polynomial time (log-log plot, left), while the pathogen in logarithmic time (log-linear plot, right). (**k**) *P*(*ξ*) vs. *ξ* as obtained from the empirical air-traffic network. We observe similar patterns to those of the random network analyzed above. Indeed, also here the *ξ*-heterogeneity ($\nu =1.4$, black solid line) drives the local efficiency levels as observed from $P({\bf{S}}\cap \xi )$ (yellow to orange, *C* = 0.01, 0.1, 0.3,1.0, 3.0, 10 days^−1^). (**l**) In decentralized mitigation, absent a distribution *network*, we find that the saved nodes are evenly spread, independently of *ξ*, an egalitarian increase in saved nodes, in which the rate heterogeneity plays no role (light to dark green, $C=0.025,\,0.03,\,0.035,\,0.04,\,0.05,\,0.1$ days^−1^).

Viruses spread via diffusion coupled with local SIR dynamics as captured by Eq. (12), Fig. 4a. In this process, upon penetration, the viruses reproduce locally at each node *n* through SIR, until reaching peak infection at $t={T}_{{\rm{Peak}},n}$. In a random network, since the majority of nodes are at the mean distance from the initial outbreak, we find that they all reach peak infection approximately simultaneously. Therefore, after a limited propagation time, the infection levels become almost homogeneous across all nodes, creating a uniformly distributed demand of the therapeutic. Indeed, we find that during the *global* peak infection, *T*_peak_, the infection distribution *P*(*j*) is bounded, capturing a state in which the majority of nodes simultaneously require treatment (Fig. 4c,e). Therefore, *when* an epidemic spreads globally, at its peak, the therapeutic *demand* is homogeneous.

#### Commodity spread

In contrast, the therapeutic follows Eq. (15), produced at a single source node *s*, then diluted as it spreads across the exponentially growing number of pathways, Fig. 4b. The result is a profoundly different spreading pattern in which the availability *q*_*n*_(*t*) follows a fat-tailed distribution, well-approximated by $P(q)\sim {q}^{-2}$ (Fig. 4d). Hence, in contrast to the homogeneous demand, *supply* is extremely heterogeneous, with a vast majority of undersupplied nodes, and a selected privileged minority of well-treated destinations (as demonstrated in Fig. 4f).

Together, this combination of *homogeneous* demand and *heterogeneous* supply, a consequence of the intrinsic spreading patterns of pathogens versus therapeutics, creates a crucial gap in our ability to achieve mitigation, as all nodes require treatment, and yet only a small minority receives sufficient supply. This discrepancy, we next show, is a practically unavoidable consequence of the network-based commodity flow underlying centralized mitigation.

### Analytical bounds for centralized mitigation

To understand the origins of the inhomogeneity observed under centralized mitigation consider the routing of the therapeutic through the network *B*_*nm*_ in Eq. (15). For every *q*_*m*_(*t*) doses present in *m*, a fraction *B*_*nm*_ will be shipped throughout the day to *n*, then yet a smaller fraction will propagate onwards to *n*’s neighbors and so on. Hence, the therapeutic availability is diluted as it flows downstream from the source *s* to the target *n* (Fig. 4b). Accounting for all pathways from *s*, the *rate* of incoming doses at *n* becomes (Supplementary Section 3.1)

6 $${\xi }_{sn}={[\mathop{\sum }\limits_{l=1}^{{L}_{{\rm{Max}}}}{B}^{l}]}_{sn},$$

namely the *s*, *n* term of a geometric series with base *B*. Roughly speaking, Eq. (6) approximates the number of doses reaching *n* per each dose exiting *s*, hence indicating which nodes benefit from superfluous drug availability (large *ξ*) and which will be underserved (small *ξ*).

In Supplementary section 3.2.2 we show that the probability density *P*(*ξ*) to observe ${\xi }_{sn}\in (\xi ,\xi +{\rm{d}}\xi )$ scales as

7 $$P(\xi )\sim {\xi }^{-\nu },$$

a power-law distribution of rates that explains the extreme levels of heterogeneity that we observed in drug supply. For a random network with an arbitrary degree-distribution we predict that $\nu =2$. This prediction, confirmed in Fig. 4g, exposes the roots of the highly unequal drug availability *P*(*q*), which, indeed, exhibits the exact same scaling in Fig. 4d.

This *ξ*-heterogenity directly impacts the probability of nodes to be *saved* by our response. A node *n* is considered saved, *i.e*. ${{\bf{S}}}_{n}=1$, if it witnesses a significant reduction in its mortality, namely that ${R}_{n}/{R}_{n,0} < {\rm{Th}}$, where *R*_*n*_ and ${R}_{n,0}$ are the long term mortality rates in *n* with and without treatment, respectively. Setting the threshold to ${\rm{Th}}=0.5$, we measured $P({\bf{S}}{\cap }^{}\xi )$, the probability that a randomly selected node in the group ${\xi }_{sn}\in (\xi ,\xi +{\rm{d}}\xi )$ has ${{\bf{S}}}_{n}=1$. As expected, we find that the greater is *ξ*_*sn*_, the larger is the probability for *n* to be saved (Fig. 4g, shades of orange). Hence, the uneven *P*(*ξ*) in (7) is, indeed, the root cause of the inefficiency observed in centralized mitigation. As *C*_*s*_ is increased, the fraction of saved nodes, namely the area under $P({\bf{S}}{\cap }^{}\xi )$, also increases, but the preference towards large *ξ*_*sn*_ continues to underlie the uneven mitigation pattern, with the saved nodes consistently concentrated around the tail of *P*(*ξ*).

The most important implications of Eq. (7) are observed through two quantities that directly impact the efficiency of centralized mitigation:

#### Critical capacity *C*_*η*_

Consider the critical capacity *C*_*η*_ required for a *successful mitigation*, defined as one where a significant fraction *η* of all nodes was saved (${{\bf{S}}}_{n}=1$). Captured by the area under the $P({\bf{S}}\cap \xi )$ curve, this maps to ${\int }_{{\xi }_{{\rm{\min }}}}^{\infty \,}P({\bf{S}}\cap \xi ){\rm{d}}\xi =\eta$, which taking *P*(*ξ*) from Eq. (7) leads to (Supplementary Section 3.2.3)

8 $${C}_{\eta }\sim {N}^{\varphi },$$

where $\varphi =1/(\nu -1)$. For a random network ($\nu =2$) this predicts ${C}_{\eta }\sim N$ (Fig. 4h). Consequently, for sufficiently large networks the critical capacity diverges polynomially with *N*, rendering efficient mitigation practically unattainable. Recall that ${C}_{s}=1$ represents a daily capacity to produce and ship sufficient supply to meet the global demand. In this sense, Eq. (8) indicates that effective mitigation requires resources that are orders of magnitude greater than the actual demand – a consequence not of the *volume* of drugs produced, but of their skewed and highly uneven distribution observed in Fig. 4d,f.

#### Mean spreading time 〈*T*〉

Last, we consider the time scales of the two competing spreads – the epidemic vs. the therapeutics. With the viruses reproducing locally at each node via SIR, their propagation times to all nodes, ${T}_{{\rm{Peak}},n}$, are determined by the length of all network paths. For a random network, in which pathways are of order^26^ ∼log*N* this predicts that, on average

9 $$\langle T\rangle =\langle {T}_{{\rm{Peak}},n}\rangle \sim \,\log \,N,$$

a rapid propagation, logarithmically dependent on system size. To evaluate the spread of the therapeutic, we seek the supply time ${T}_{{\rm{Supp}},n}$, as the time when ${q}_{n}(t={T}_{{\rm{Supp}},n})=1$, *i.e*. the time when* n*’s local demand has been met. Once again, the power-law distribution of (7) predicts that the mean supply time follows (Supplementary Section 3.2.4)

10 $$T=\langle {T}_{{\rm{Supp}},n}\rangle \sim {N}^{\varphi },$$

a much slower propagation that diverges with the system size. In Fig. 4i,j we show 〈*T*〉 vs. *N* for the epidemic (red) and the therapeutics (blue). As predicted, the two spreading processes are characterized by different spreading times – logarithmic vs. polynomial – ensuring that for sufficiently large *N*, we have $\langle {T}_{{\rm{Supp}},n}\rangle \gg \langle {T}_{{\rm{Peak}},n}\rangle$, namely that supply is guaranteed to lag significantly behind demand.

Equations (7–10) expose an intrinsic lacuna of centralized mitigation, that, by virtue of relying on a network-based distribution scheme, it leads to an extremely unequal distribution, and hence to a highly discriminative spread of the therapeutic. This inequality directly translates to the observed mitigation inefficiency. Our analysis is independent of disease/commodity flow parameters. These may affect the specific rates and pre-factors, but have little bearing on the scaling exponents, which are, indeed, intrinsic to the spreading dynamics. Therefore, as long as the epidemic spreads globally – a scenario often considered imminent - centralized mitigation requires prohibitive manufacturing and shipping capacities (Fig. 4h); in their absence – the epidemic will inevitably outrun the therapeutics (Fig. 4i,j). We, therefore, find that the only remedy is to design a non-network-based dissemination scheme, *i.e*. decentralized mitigation.

### Application to empirical networks

To examine *P*(*ξ*) in real distribution networks, we constructed *B*_*nm*_ from empirical fluxes of human mobility (Supplementary Section 2.2) and used (6) to obtain the incoming shipping rates *ξ*_*sn*_ of all nodes *n*. We find, in Fig. 4k that *P*(*ξ*) indeed follows the power-law of (7), here with $\nu =1.4$, an extremely uneven distribution, where most nodes receive just a tiny fraction of the therapeutic exiting *s*. We also measured $P({\bf{S}}\cap \xi )$, finding again that the saved nodes are concentrated around the large *ξ* tail of *P*(*ξ*), confirming that network dilution via (6) is, indeed, the source of the unsuccessful mitigation. Repeating this experiment for decentralized mitigation (Fig. 4l) shows the intrinsic difference between the two strategies, as here, since *B*_*nm*_ plays no role in delivering the therapeutic, $P({\bf{S}}\cap \xi )$ rises uniformly across all nodes, independently of their highly uneven pathways to the source *s*.

While our analytical predictions focus on random networks, we have shown that they also apply to the empirical *B*_*nm*_, which builds on the *natural* fluxes, as extracted from global aviation data^27,28^. More broadly, such dilution in the downstream flow of the therapeutic in the form of Eq. (6), is an intrinsic consequence of any reasonable network construction^29^, and hence we believe that it is practically unavoidable under the centralized mitigation framework. In Supplementary Section 2.2 we artificially construct an *egalitarian network*, which indeed rectifies, to some extent, the observed distribution inequality. However, the realization of such a network requires us to seize full control over air transportation, which is not only an unrealistic scenario, but also, as mentioned above, one that imposes an extremely heavy burden on the global economy and political stability.

### Optimizing centralized mitigation

**Figure 5:**
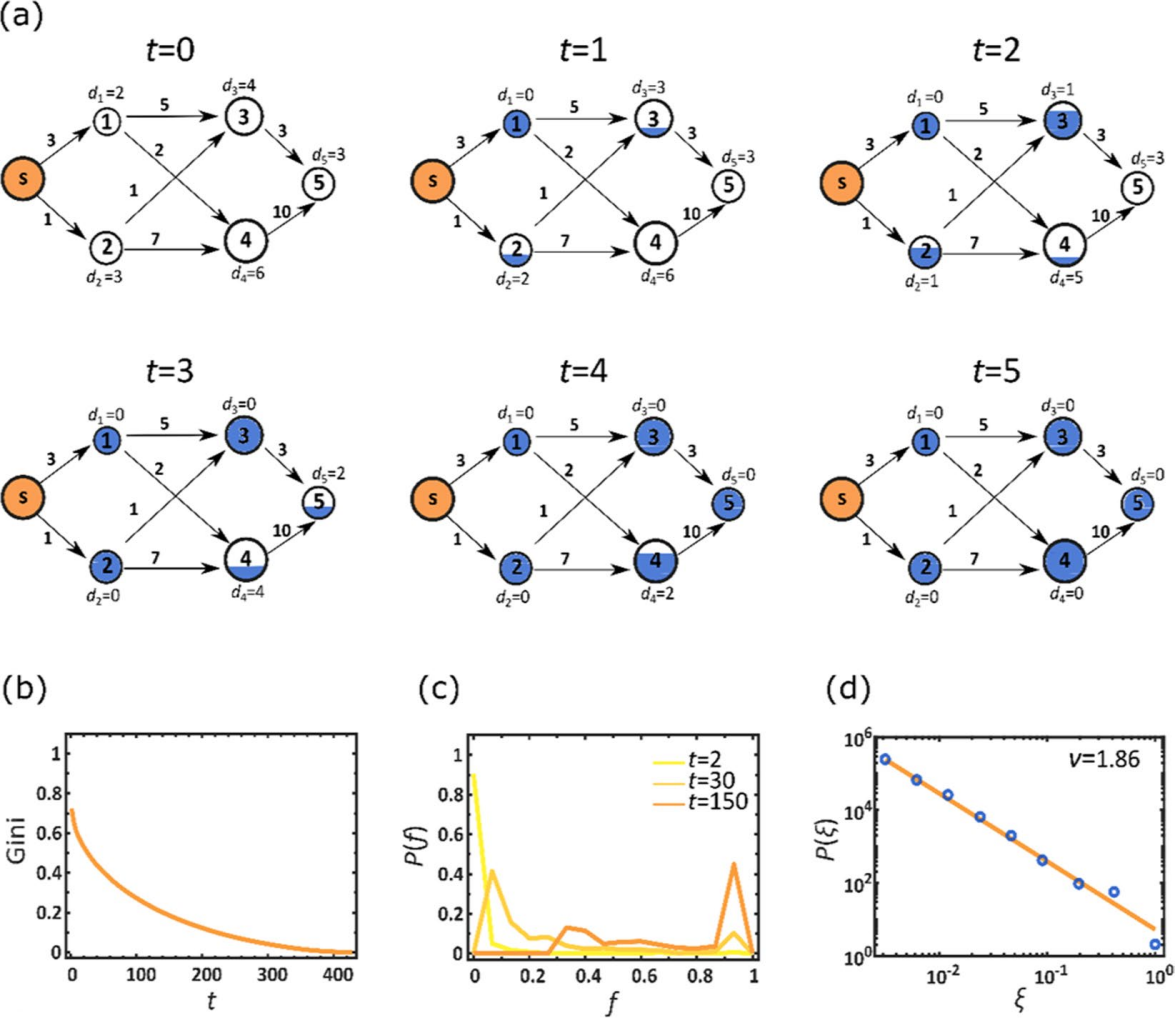
Optimizing centralized mitigation. (**a**) To enhance the efficiency of the centralized drug distribution we used the commodity flow framework: each node is assigned an initial demand *d*_*n*_(0), proportional to its population (node size); and each air-route is assigned a daily carrying capacity *C*_*nm*_ (numbers by edges), proportional to its daily volume of passenger traffic. In each time step *t* we optimize the flow from the source *s* to supply as much of the therapeutic as possible, within the constrained *C*_*nm*_. As nodes accumulate the therapeutic (blue fill) their demand is updated accordingly. For example, node 2’s initial demand is *d*_2_(0) = 3. Following the first round of shipment (*t* = 1) a single unit is shipped from *s*, supplying a fraction ${f}_{2}(1)=1/3$ of *d*_2_(0), and hence reducing its demand to *d*_2_(1) = 2. A node is fully supplied (fully blue) at time ${T}_{{\rm{Supp}},n}$, when ${f}_{n}(t={T}_{{\rm{Supp}},n})=1,\,\,$and accordingly ${d}_{n}({T}_{{\rm{Supp}},n})=0$. For node 2 we have ${T}_{{\rm{Supp}},2}=3$. (b) The Gini coefficient vs. *t*, as obtained from the time dependent fractional stock levels *f*_*n*_(*t*). We find that despite the optimization, inequality continues to govern the therapeutic distribution, as expressed by the high Gini coefficient at small *t*. This describes a scenario in which few nodes are fully stocked early on, while the majority of nodes will only reach ${f}_{n}(t)\to 1$ (and hence Gini → 0) at much later times. (**c**) The probability density *P*(*f*) vs. *f* at three selected time points *t*. Similarly to Fig. 3b we observe a highly unequal distribution in which selected nodes are fully supplied (right peaks), while others are still deprived (left peaks). These patterns in (**b**) and (**c**) are strikingly reminiscent of the those observed earlier in Fig. 3a,b. (**d**) *P*(*ξ*) vs. *ξ*, capturing the probability density that a randomly selected node enjoys a supply rate of $\xi \in (\xi +{\rm{d}}\xi )$. Here we approximate rates as ${\xi }_{sn}=1/{T}_{{\rm{Supp}},n}$, namely the average supply rate in *n* under therapeutic distribution from *s*. The power-law form of *P*(*ξ*) (solid line represents ${\xi }^{-\nu }$) demonstrates the uneven dissemination of the therapeutic, in which supply rates range over orders of magnitude. This pattern of *P*(*ξ*) is, again, analogous with the identical patterns of distribution inequality observed in Fig. 4d,g,k, further reinforcing the intrinsic heterogeneity characterizing network-based centralized mitigation.

Our modeling of centralized mitigation up to this point was based on diffusive spread, *a la* Eq. (15), a framework that can be naturally coupled to the SIR dynamics of Eq. (12). In this framework, our control over the dissemination is enacted through the design of the networks *B*_*nm*_ and *Z*_*nm*_ (Supplementary Section 2.2). Once these networks are set, the spread of the therapeutic is governed by diffusion, which is often sub-optimal, allowing, *e.g*., for superfluous quantities to accumulate at selected locations. We can improve dissemination efficiency by modeling it as a commodity flow problem^30,31^, seeking to optimally utilize the routes of the existing air-traffic network, until meeting the demands of all destinations^32,33^. In this framework, each air-route is assigned a carrying capacity *C*_*nm*_, capturing the number of doses it *can* transport per day, and each destination *n* is assigned an initial demand *d*_*n*_(0), depending on the size of its local population *M*_*n*_. At each step (day), as the therapeutic is shipped and accumulates at *n*, the local demands are updated, ${d}_{n}(t)$, subtracting the supplied doses from *d*_*n*_(0), until *n*’s quota is filled at time ${T}_{{\rm{Supp}},n}$ (Fig. 5a). Using linear optimization, we derive the optimal dissemination strategy to achieve maximum daily flow to all destinations $n=1,\ldots ,N$ from the source *s*, avoiding any wasted dosage via oversupply, and satisfying the constrained carrying capacities *C*_*nm*_ (Supplementary Section 5).

Our previous analysis in Figs 3 and 4 indicated that the main problem in centralized dissemination is its extreme levels of inequality, as expressed through the efficiency ${{\epsilon }}_{{\rm{Res}},n}$ in Eq. (4). In the context of the current modeling this is most naturally expressed through

11 $${f}_{n}(t)=1-\frac{{d}_{n}(t)}{{d}_{n}(0)},$$

capturing the fraction of *n*’s demand that is supplied by the time *t*. Interestingly, we find in Fig. 5 that the optimal commodity flow, in spite of being profoundly different from the diffusive propagation of Eq. (15), leads to strikingly similar patterns of inhomogeneity. For instance, the Gini coefficient extracted from *f*_*n*_(*t*) remains large at the early stages of the dissemination (Fig. 5b), reminiscent of the patterns observed for ${{\epsilon }}_{{\rm{Res}},n}$ in Fig. 3a above. This indicates that few nodes fill their initial demand early on, while the majority of nodes take a long time to satisfy their quota, hence the large inequality observed for small *t*. Similar patterns are also observed through the time evolution of *P*(*f*), capturing the probability density for a random node *n* to have ${f}_{n}(t)\in (f,f+{\rm{d}}f)$. Indeed, Fig. 5c shows that *P*(*f*) recovers the signature two peak structure observed earlier for $P({{\epsilon }}_{{\rm{Res}}})$: an increased density around $f\to 0$ and $f\sim 1$, capturing a coexistence of early vs. late supplied nodes (compare to Fig. 3b). Finally, we used ${T}_{{\rm{Supp}},n}$, the time for *n* to fill its demand, to estimate *n*’s *average supply rate* as ${\xi }_{sn}=1/{T}_{{\rm{Supp}},n}$, namely the average volume of doses entering *n* per unit time. In Fig. 5d we find that *P*(*ξ*) recovers the power-law form predicted in (7) with $\nu =1.86$, hence fully retrieving the patterns of distribution inequality exposed in Fig. 4g,k.

Together we find that even under optimal distribution, the unequal supply rate, indeed the root cause of inefficiency of centralized mitigation, is practically impossible to avoid. Therefore, it is not unique to our modeling via Eq. (15), or to our specific network design, but rather represents an intrinsic characteristic of network-based dissemination, further illustrating the crucial need for a decentralized mitigation strategy.

Box 1. Digitizable therapeutics While classic medications are shipped and transported in their physical form, a *digitizable* therapeutic can be distributed as data and manufactured on location via scalable printing technology. We consider three relevant therapeutic media with digitizable potential:

**Small molecules**. Envisioned as a future means of drug distribution, technology for printing biomedically relevant molecules has been recently introduced. The molecular 3D printers are designed to enable several pharmaceutical applications, such as drug design, personalized drug dosing and the fabrication of treatment devices^41–43^. The main advantage of small molecules is that they underlie the majority of current mainstream pharma. Indeed, most established drugs are, at present, based on small molecules, and are therefore considered the natural candidate for treating pathogens.

**Nucleic acids**. Built from a selection of four natural building blocks (G, C, A, T/U) and additional artificial ones, their synthesis is sufficiently facile and inexpensive as to be carried out locally^16,18^. DNA/RNA sequences operate either by interfering with gene expression, *e.g*., antisense oligonucleotides^44–46^ and RNAi^47–49^, or through their unique folding geometry, which allows them to interact structurally with a target molecule, *e.g*., aptamers^16,50–54^ and ribozymes^55–57^. While not widespread at this point, these short DNA-sequences have already proven their potential efficacy, for example inhibiting the activity of human immunodeficiency virus (HIV)^58,59^, as well as other applications at various stages of clinical trials^52^. A crucial advantage of DNA aptamers is their rapid *ab-initio* discovery, which can be accomplished within hours or days^60^, allowing a potentially rapid response when confronted with an unknown pathogen.

**Peptides**. Peptide sequences are coded from a selection of twenty natural building blocks, *i.e*. amino acids, folding into a defined geometric 3D structure to bind or modify selected target molecules^61–63^. Short peptides can be efficiently digitized, thanks to their relatively facile printability^64^; larger ones, however, often require additional modification^65^, and are hence harder to produce and fold properly. Finally, proteins, essentially very large peptides, are at present too complex for local synthesis outside a biological system, such as a cell culture, therefore prohibiting their current use as locally synthesized therapeutics.

The main advantage of nucleic acids and peptides is their polymeric structure, comprising a limited set of discrete recurring building blocks. These building blocks are *digital* in the same sense that binary data is *digital*, with the sequence of monomers serving as data *bits*. Consequently, one can flexibly print any desired sequence using a standard printing apparatus, without specific adaptation or calibration, in a similar fashion to the way a standard printer can print any desired text. In the face of a novel pathogen, such flexibility can provide a crucial advantage over molecular printing. The therapeutic potential of such polymer-based drugs, while not yet widespread, has been demonstrated on several existing treatments, such as Fomivirsen^66^, Pegaptanib^67^ and Insulin^68^. Their digital advantage, we believe, merits further research into expanding their therapeutic applicability.

Box 2. Modeling a lethal global epidemic under centralized and decentralized mitigation In a network of *N* coupled nodes $n=1,\ldots ,N$, each with a population of *M*_*n*_ individuals, we use the SIR model to track the fraction of *M*_*n*_ who are susceptible (*s*_*n*_), infected (*j*_*n*_) or removed (*r*_*n*_). Each of these sub-populations is divided among the treated individuals (${s}_{n}^{{\rm{T}}},\,{j}_{n}^{{\rm{T}}},\,{r}_{n}^{{\rm{T}}}$), who have been provided a therapeutic, and the untreated individuals (${s}_{n}^{{\rm{U}}},\,{j}_{n}^{{\rm{U}}},\,{r}_{n}^{{\rm{U}}}$), who have not yet gained access to it. For a lethal epidemic, ${r}_{n}^{{\rm{U}}}$ represents the deceased population, while ${r}_{n}^{{\rm{T}}}$ are the *saved* individuals, who, absent any treatment, would have perished. The epidemic dynamics is driven by (Supp. Sec. 1)

$$\frac{{\rm{d}}{s}_{n}^{{\rm{U}}}}{{\rm{d}}t}=-\,\alpha {s}_{n}^{{\rm{U}}}{j}_{n}\sigma ({j}_{n})-\gamma \rho ({q}_{n}){s}_{n}^{{\rm{U}}}+\mathop{\sum }\limits_{m=1}^{N}{A}_{nm}({s}_{m}^{{\rm{U}}}-{s}_{n}^{{\rm{U}}})$$

$$\frac{{\rm{d}}{s}_{n}^{{\rm{T}}}}{{\rm{d}}t}=-\,\alpha {s}_{n}^{{\rm{T}}}{j}_{n}\sigma ({j}_{n})+\gamma \rho ({q}_{n}){s}_{n}^{{\rm{U}}}+\mathop{\sum }\limits_{m=1}^{N}{A}_{nm}({s}_{m}^{{\rm{T}}}-{s}_{n}^{{\rm{T}}})$$

$$\frac{{\rm{d}}{j}_{n}^{{\rm{U}}}}{{\rm{d}}t}=\alpha {s}_{n}^{{\rm{U}}}{j}_{n}\sigma ({j}_{n})-\beta {j}_{n}^{{\rm{U}}}-\gamma \rho ({q}_{n}){j}_{n}^{{\rm{U}}}+\mathop{\sum }\limits_{m=1}^{N}{A}_{nm}({j}_{m}^{{\rm{U}}}-{j}_{n}^{{\rm{U}}})$$

$$\frac{{\rm{d}}{j}_{n}^{{\rm{T}}}}{{\rm{d}}t}=\alpha {s}_{n}^{{\rm{T}}}{j}_{n}\sigma ({j}_{n})-\zeta {j}_{n}^{{\rm{T}}}+\gamma \rho ({q}_{n}){j}_{n}^{{\rm{U}}}+\mathop{\sum }\limits_{m=1}^{N}{A}_{nm}({j}_{m}^{{\rm{T}}}-{j}_{n}^{{\rm{T}}})$$

$$\frac{{\rm{d}}{r}_{n}^{{\rm{U}}}}{{\rm{d}}t}=\beta {j}_{n}^{{\rm{U}}}$$

12 $$\frac{{\rm{d}}{r}_{n}^{{\rm{T}}}}{{\rm{d}}t}=\zeta {j}_{n}^{{\rm{T}}}+\mathop{\sum }\limits_{m=1}^{N}{A}_{nm}({r}_{m}^{{\rm{T}}}-{r}_{n}^{{\rm{T}}})$$

where *α* is the infection rate, *β* is the mortality rate of the untreated individuals and $\zeta  > \beta$ is the recovery rate under treatment. The therapeutic efficacy is $0\le \gamma \le 1$ and its consumption rate $\rho ({q}_{n})$ depends on the availability of the therapeutic *q*_*n*_(*t*) in *n*, as

13 $$\rho ({q}_{n})=\,{\rm{\min }}\{\frac{{q}_{n}(t)}{{s}_{n}^{{\rm{U}}}(t)+{j}_{n}^{{\rm{U}}}(t)},1\}.$$

Hence $\rho ({q}_{n})$ increases linearly with *q*_*n*_(*t*) as long as the demand (denominator) exceeds the supply, and saturates to unity when *n* has excess quantities of the therapeutic, avoiding over consumption (Supp. Sec. 1.2). In (12) we introduce an invasion threshold *ε* through the sigmoidal function

14 $$\sigma ({j}_{n})=\frac{{({j}_{n}/\varepsilon )}^{h}}{{({j}_{n}/\varepsilon )}^{h}+1},$$

which activates the local SIR dynamics only when the local infection levels ${j}_{n}={j}_{n}^{{\rm{U}}}+{j}_{n}^{{\rm{T}}}$ exceed *ε*. The diffusion of individuals between nodes is mediated by *A*_*nm*_, derived from the empirical international air-travel network^61^ (Supp. Sec. 2.1).

**Drug availability**. Under *Centralized mitigation* we have

15 $$\frac{{\rm{d}}{q}_{n}}{{\rm{d}}t}=-\,\rho ({q}_{n})({s}_{n}^{{\rm{U}}}+{j}_{n}^{{\rm{U}}})+\theta (t-{t}_{{\rm{R}}}){\delta }_{ns}{\kappa }_{s}{C}_{s}+\mathop{\sum }\limits_{m=1}^{n}({Z}_{nm}{q}_{m}-{B}_{mn}{q}_{n}),$$

where the first term captures local drug consumption, and the second term, activated following a response time *t*_R_, represents drug production/shipment from the source *s*; *δ*_*ns*_ is the Kronecker *δ*-function and $\theta (x)$ is the Heavyside step-function. The pre-factor ${\kappa }_{s}$ is derived in Supp. Sec. 1.3. The shipping routes are governed by *Z*_*nm*_ and *B*_*nm*_, constructed in Supp. Sec. 2.2.

In Supp. Sec. 5 we also consider optimized distribution strategies based on commodity flow algorithms.

In *Decentralized mitigation* we have

16 $$\frac{{\rm{d}}{q}_{n}}{{\rm{d}}t}=-\rho ({q}_{n})({s}_{n}^{{\rm{U}}}+{j}_{n}^{{\rm{U}}})+\theta (t-{t}_{{\rm{R}}}){c}_{n}$$

in which the central production in *s* is replaced by local production in each node at a rate *c*_*n*_. Here $\langle {c}_{n}\rangle ={C}_{s}=C$ translates to a cumulative production capacity of a *C*-fraction of the global demand per day, with the only distinction being whether this production is centralized (*C*_*s*_) or decentralized (*c*_*n*_); Supp. Sec. 1.3.

## Discussion

Network spreading processes are at the heart of many crucial applications, from the flow of information to the diffusion of physical commodities. The resulting propagation patterns may be highly diverse, owing to the distinct spreading dynamics governing each process^34,35^. The consequences for disease mitigation are crucial, as we find that diseases spread roughly homogeneously, while therapeutics tend to distribute extremely heterogeneously. Intrinsic to the nature of commodity vs. viral flow these patterns are practically impossible to avoid – placing severe limits on our ability to efficiently address global epidemics. We, therefore introduce decentralized mitigation, a currently unexplored strategy, as likely the only tenable response for this threat.

While current technology is not fully mature for immediate implementation of decentralization, in Section A we discuss its potential applicability within the foreseeable future, providing estimates for the level of technological enhancement that it entails. We also emphasize that in real scenarios, a combined approach is likely best, where highly capable populations (*c*_*n*_ > 1) can ship their excess synthesized therapeutics to less capable ones (*c*_*n*_ < 1). This hybrid – physical/digital – strategy can further homogenize the therapeutic supply, and minimize the burden on the transportation networks to treat only the needy destinations. Hence, even if imperfect, decentralization capabilities are a crucial component of future mitigation of global pandemics.

Our findings are most crucial in case the epidemic spreads globally. In such scenario, the peak infection occurs approximately simultaneously at logarithmic time-scales. Equations (8) and (10) indicate that such concurrent global demand cannot be met even under the most optimistic estimates for the capacity *C*_*s*_. Under these circumstances, decentralized mitigation is more efficient, even if it is inferior in other characteristics, such as production rate (*c*_*n*_) or efficacy (*γ*). This calls for a change in the current paradigm of classification and prioritization of therapeutics. At present, we focus mainly on the therapeutic efficacy^36,37^, *i.e*. how efficiently the biological/chemical agent cures the disease. We relate little weight, however, to the agent’s chemical classification – *e.g*., whether it is a small molecule, a protein, or a nucleic acid, as, indeed, these details seem marginal as long as it overcomes the lethal pathogen. However, these distinctions become crucial if we consider *digital-distributability*, as not all molecular media are equally digitizable. Hence, the utility of a drug emerges not just from its chemical effectiveness, but also from how such effectiveness is balanced alongside the drug’s distributability.

Most crucially, our analysis shows that *mitigation effectiveness*, *i.e*. the amount of lives saved, may be distinct from therapeutic efficacy. This motivates future research into decentralizable therapeutic technologies, which, even if biomedically inferior, may translate to significantly more lives saved under a global spreading scenario (see Box 1 and Section A).

In a broader perspective, our finding that homogenous spread enhances mitigation efficiency may be relevant in other distribution scenarios, even if digitizablity is not available. For instance, most distribution algorithms for physical commodities optimize for maximal flow or for minimum cost^30–32,38–40^. However, our results indicate that optimizing for homogeneity may often provide the most desirable outcome, not only in terms of equality, but also in terms of overall mitigation efficiency.

### Section A - biological applicability and current gaps

Decentralization is motivated primarily by network science considerations, showing that it enhances equality and hence the mitigation efficiency. Its realization, however, is limited by bio-technology, which at present offers several pathways towards digitization, as discussed in Box 1. Here we analyze the challenges in the DNA aptamer path, as we believe it exhibits several advantages^16^ over the alternatives, such as the diversity of their initial random library^69^ their rapid discovery process^60^, and their relatively stable nature, which underlies their smooth handling and shipping, compared to, *e.g*., peptides. We are also motivated by recent reviews^16,70^ that expose the therapeutic potential of DNA aptamers, citing the major obstacle towards their biomedical applicability as the lagging research rather than any intrinsic therapeutic deficiency they possess. Of course, other biomedical realizations are equally relevant, as our main focus is on the efficiency of decentralization as a *mitigation strategy*, not on advocating for a specific biomedical application.

The main bottleneck on the track to application via DNA-based therapeutics is the scalability of DNA printing^16^. Present day oligonucleotide synthesizers are capable of synthesizing ∼10 gram/day of a short oligonucleotide, at a cost of ∼500 USD, requiring thorough purification, and generating, as a byproduct, large amounts of liquid waste. For RNA nucleosides, LNA, or other modified materials, the cost becomes approximately 10 times higher, *i.e*. ∼5,000 USD per 10 grams. Combined with the current cost of the required instrumentation, estimated at ∼2.5 × 10^5^ USD, we believe, that despite the unequivocal advantages of decentralization, it is currently of limited applicability at a global scale. This status-quo, however, is a consequence of our current priorities, rather than an intrinsic technological restriction. Indeed, the need for scalable low-cost mass-printing of short DNA sequences was not evident until now, and hence the development of the relevant technology was never prioritized. Our findings, together with the potential usefulness of such therapeutics^16,70^, suggest reassessing these priorities.

Another issue regarding the mass-production of DNA-based therapeutics, is the need for large amounts of phosphorous, a limited resource, that constitutes ∼10% of the DNA mass^71^. It was recently estimated that Earth’s accessible phosphorous reserves are in excess of 6 × 10^13^ kg, with global production in 2016 amounting to 2.6 × 10^11^ kg, mainly serving the fertilizer industry^72^. Our calculations, based on these figures, indicate that phosphorous availability is orders of magnitude higher than that required for global dissemination of our proposed DNA-based therapeutics. To be on the safe side, we assume the oligonucleotide dosage to be of the order of ∼10^−2^ kg per person, and the synthesis yield to be only 10%. Even under these stringent assumptions, we can treat the global population of ∼10^10^ individuals with ∼10^8^ kg of phosphorous, amounting to less than 0.1% of the current annual consumption.

Most crucially, our findings clearly show that even imperfect digitizable therapeutics – *e.g*., costly or non-efficient (low *c*_*n*_), are still significantly more effective than the non-digitizable (centralized) alternative. Our analysis indicates that *c*_*n*_ ∼ 0.2, a distributed coverage of approximately 20%, can efficiently tackle a virulent epidemic. Hence, to implement decentralized mitigation capabilities we must aim for local production capacity of the order of 2 × 10^5^ doses per day – printing and locally disseminating – per each population of 10^6^ individuals. At ∼1 gram per dose, this requires ∼2 × 10^4^ printers with a synthesis rate of ∼10 grams/day each. Under current costs, this amounts to ∼5 × 10^3^ USD per individual. Therefore, to meet this desired capacity, we must aim for a 10–100 fold reduction in the cost of the required instrumentation. Once achieved, the infrastructure for decentralized mitigation can be established over the course of ∼10 years with an annual investment of 5–50 USD per person. While a 10–100 fold cost reduction is, indeed, no incremental advance, we believe that it is within our reach if only the priority is set.

## Supplementary information

Supplementary Information

## Data Availability

Numerical codes to reproduce the results presented in the paper are available at https://figshare.com/projects/Digitable_therapeutics_for_decentralized_mitigation_of_global_pandemics/69062.
