# Supplementary material for: Digitizable therapeutics for decentralized mitigation of global pandemics
Source: Sci Rep. 2019 Oct 4;9:14345. doi: 10.1038/s41598-019-50553-x (PMC6778202; doi:10.1038/s41598-019-50553-x)
Supplement: Supplementary file 1 — Supplementary Information [file 41598_2019_50553_MOESM1_ESM.pdf]

Digitizable therapeutics for decentralized mitigation of global pandemics

Supplementary Material

Adar Hacohen, Reuven Cohen, Sol Efroni, Baruch Barzel & Ido Bachelet

Contents

1 Modeling epidemic spread **1**
1.1 SIR model . . . 1
1.2 SIR model with treatment . . . 2
1.3 Normalized equations . . . 4
1.4 The human flux matrix . . . 7

2 Implementation **9**
2.1 Human mobility network and epidemic parameters . . . 9
2.2 Drug distribution networks . . . 10

3 Heterogeneity in network-based distribution **14**
3.1 The rate matrix ξ_{sn} . . . 14
3.2 Supply patterns in random networks . . . 15
3.2.1 Network pathways . . . 15
3.2.2 Rate distribution $P(\xi)$ . . . 18
3.2.3 Critical capacity C_η . . . 19
3.2.4 Mean supply time $\langle T \rangle$ . . . 20

4 Extended testing **21**
4.1 The effect of the therapeutic distribution source . . . 21
4.2 The effect of disease parameters . . . 22

5 Optimized physical distribution **22**
5.1 Overview . . . 22
5.2 Maximum flow optimization . . . 25

1 Modeling epidemic spread

1.1 SIR model

To model epidemic spreading via air travel [1] we used a local susceptible-infected-recovered (SIR) model with diffusive coupling, following Ref. [2]. In this framework each node n ($n = 1, \ldots, N$) represents a local population of M_n individuals, of which $S_n(t)$ are in the susceptible state $\mathcal{S}$, $I_n(t)$ are infected ($\mathcal{I}$) and $R_n(t)$ are recovered ($\mathcal{R}$), hence $S_n(t) + I_n(t) + R_n(t) = M_n$ for all t. Within each node, we assume a well-mixed population that locally follows SIR dynamics [3–6], namely

$$\begin{aligned} \mathcal{I} + \mathcal{S} &\stackrel{\alpha}{\to} 2\mathcal{I} \\ \mathcal{I} &\stackrel{\beta}{\to} \mathcal{R}, \end{aligned} \tag{1.1}$$

where α and β are the infection and recovery rates, respectively. The coupling between two populations n and m is mediated by the flux of incoming/outgoing travelers via air-traffic F_{nm}, quantifying the number of individuals flying from m to n per day.

This form of epidemic spreading gives rise to the following dynamic equations [2, 7]

$$\frac{\mathrm{d}S_n}{\mathrm{d}t} = -\alpha \frac{S_n(t) I_n(t)}{M_n} + \sum_{m=1}^{N} \Big(w_{nm} S_m(t) - w_{mn} S_n(t) \Big) \tag{1.2}$$

$$\frac{\mathrm{d}I_n}{\mathrm{d}t} = \alpha \frac{S_n(t) I_n(t)}{M_n} - \beta I_n(t) + \sum_{m=1}^{N} \Big(w_{nm} I_m(t) - w_{mn} I_n(t) \Big) \tag{1.3}$$

$$\frac{\mathrm{d}R_n}{\mathrm{d}t} = \beta \mathcal{I}_n(t) + \sum_{m=1}^{N} \Big(w_{nm} R_m(t) - w_{mn} R_n(t) \Big). \tag{1.4}$$

The first term/s on the r.h.s. capture the processes of infection, proportional to the product of susceptible and infected individuals, and recovery, proportional to $I_n(t)$. The summation terms describe the diffusion of $\mathcal{S}, \mathcal{I}$ or $\mathcal{R}$ individuals between local populations, where

$$w_{nm} = \frac{F_{nm}}{M_m} \tag{1.5}$$

is the per-capita flux from m to n, hence, *e.g.*, $w_{nm} S_m(t)$ is the volume of susceptible passengers leaving m and entering n per day.

Finally, we introduce an invasion threshold ε, which *activates* the local SIR dynamics only if the infected population rises above an ε fraction of the local population. We apply this by adding a sigmoidal function [2]

$$\sigma(x) = \frac{(x/\varepsilon)^h}{1 + (x/\varepsilon)^h} \tag{1.6}$$

to the relevant equation terms, providing

$$\frac{\mathrm{d}S_n}{\mathrm{d}t} = -\alpha\frac{S_n(t)I_n(t)}{M_n}\sigma\left(\frac{I_n(t)}{M_n}\right) + \sum_{m=1}^{N}\Big(w_{nm}S_m(t) - w_{mn}S_n(t)\Big) \tag{1.7}$$

$$\frac{\mathrm{d}I_n}{\mathrm{d}t} = \alpha\frac{S_n(t)I_n(t)}{M_n}\sigma\left(\frac{I_n(t)}{M_n}\right) - \beta I_n(t) + \sum_{m=1}^{N}\Big(w_{nm}I_m(t) - w_{mn}I_n(t)\Big) \tag{1.8}$$

$$\frac{\mathrm{d}R_n}{\mathrm{d}t} = \beta I_n(t) + \sum_{m=1}^{N}\Big(w_{nm}R_m(t) - w_{mn}R_n(t)\Big), \tag{1.9}$$

hence *infection* is locally initiated only when $I_n(t)/M_n$ exceeds ε, an *invasion* of n.

1.2 SIR model with treatment

We now consider drug distribution, which divides the population into two separate groups, the treated population, who received the drug $\mathcal{Q}$, and the untreated population who have not yet acquired it. This leads to six distinct states, characterizing each node's population:

$$\mathcal{S}^{\mathrm{U}}, \mathcal{I}^{\mathrm{U}}, \mathcal{R}^{\mathrm{U}}, \tag{1.10}$$

the untreated individuals who are susceptible, infected or recovered, vs.

$$\mathcal{S}^{\mathrm{T}}, \mathcal{I}^{\mathrm{T}}, \mathcal{R}^{\mathrm{T}} \tag{1.11}$$

the treated individuals in each state. The possible transitions in each node are now driven by

$$\begin{array}{rcl}
\mathcal{S}^{\mathrm{U}} + \mathcal{Q} & \xrightarrow{\gamma\rho} & \mathcal{S}^{\mathrm{T}} \\
\mathcal{I}^{\mathrm{U}} + \mathcal{Q} & \xrightarrow{\gamma\rho} & \mathcal{I}^{\mathrm{T}} \\
\mathcal{S}^{\mathrm{U}} + \mathcal{Q} & \xrightarrow{(1-\gamma)\rho} & \mathcal{S}^{\mathrm{U}} \\
\mathcal{I}^{\mathrm{U}} + \mathcal{Q} & \xrightarrow{(1-\gamma)\rho} & \mathcal{I}^{\mathrm{U}} \\
\mathcal{I}^{\mathrm{U}} + \mathcal{S}^{\mathrm{U}} & \xrightarrow{\alpha} & 2\mathcal{I}^{\mathrm{U}} \\
\mathcal{I}^{\mathrm{T}} + \mathcal{S}^{\mathrm{U}} & \xrightarrow{\alpha} & \mathcal{I}^{\mathrm{T}} + \mathcal{I}^{\mathrm{U}} \\
\mathcal{I}^{\mathrm{U}} + \mathcal{S}^{\mathrm{T}} & \xrightarrow{\alpha} & \mathcal{I}^{\mathrm{U}} + \mathcal{I}^{\mathrm{T}} \\
\mathcal{I}^{\mathrm{T}} + \mathcal{S}^{\mathrm{T}} & \xrightarrow{\alpha} & 2\mathcal{I}^{\mathrm{T}} \\
\mathcal{I}^{\mathrm{U}} & \xrightarrow{\beta} & \mathcal{R}^{\mathrm{U}} \\
\mathcal{I}^{\mathrm{T}} & \xrightarrow{\zeta} & \mathcal{R}^{\mathrm{T}},
\end{array} \tag{1.12}$$

where ρ is the drug consumption rate, β is the recovery rate from the untreated disease, as appears in (1.1), and $\zeta > \beta$ is the recovery rate under treatment. The drug efficacy is $0 \le \gamma \le 1$, capturing the probability of recovery (at rate ζ) following drug consumption, hence for each consumed dose, at a rate ρ, a fraction γ of the consumers transitions to the treated state, while a $1-\gamma$ fraction remains untreated. In other words, the first four *reactions* represent the process of *treatment*, in which a single dose $\mathcal{Q}$ is consumed and an untreated individual transitions with probability γ to the treated state. For a fatal

disease, the untreated population perishes, hence $R_n^{\mathrm{U}}(t)$, the number of individuals at the $\mathcal{R}^{\mathrm{U}}$ state in n represents n's deceased population, while $R_n^{\mathrm{T}}(t)$, counting individuals in the $\mathcal{R}^{\mathrm{T}}$ state, represents the number of saved individuals, who would, in the absence of the therapeutic, also perish. In (1.12) we assume preemptive drug dissemination, in which once the therapeutics become available, they are provided to the entire population - susceptible or infected - such that a susceptible individual can undergo treatment directly upon infection, without the additional lag for acquiring the therapeutic.

We arrive at the following six equations

$$\frac{\mathrm{d}S_n^{\mathrm{U}}}{\mathrm{d}t} = -\alpha\frac{S_n^{\mathrm{U}}I_n}{M_n}\sigma\left(\frac{I_n}{M_n}\right) - \rho\gamma S_n^{\mathrm{U}} + \sum_{m=1}^{N}\left(w_{nm}S_m^{\mathrm{U}} - w_{mn}S_n^{\mathrm{U}}\right) \tag{1.13}$$

$$\frac{\mathrm{d}S_n^{\mathrm{T}}}{\mathrm{d}t} = -\alpha\frac{S_n^{\mathrm{T}}I_n}{M_n}\sigma\left(\frac{I_n}{M_n}\right) + \rho\gamma S_n^{\mathrm{U}} + \sum_{m=1}^{N}\left(w_{nm}S_m^{\mathrm{T}} - w_{mn}S_n^{\mathrm{T}}\right) \tag{1.14}$$

$$\frac{\mathrm{d}I_n^{\mathrm{U}}}{\mathrm{d}t} = \alpha\frac{S_n^{\mathrm{U}}I_n}{M_n}\sigma\left(\frac{I_n}{M_n}\right) - \beta I_n^{\mathrm{U}} - \rho\gamma I_n^{\mathrm{U}} + \sum_{m=1}^{N}\left(w_{nm}I_m^{\mathrm{U}} - w_{mn}I_n^{\mathrm{U}}\right) \tag{1.15}$$

$$\frac{\mathrm{d}I_n^{\mathrm{T}}}{\mathrm{d}t} = \alpha\frac{S_n^{\mathrm{T}}I_n}{M_n}\sigma\left(\frac{I_n}{M_n}\right) - \zeta I_n^{\mathrm{T}} + \rho\gamma I_n^{\mathrm{U}} + \sum_{m=1}^{N}\left(w_{nm}I_m^{\mathrm{T}} - w_{mn}I_n^{\mathrm{T}}\right) \tag{1.16}$$

$$\frac{\mathrm{d}R_n^{\mathrm{U}}}{\mathrm{d}t} = \beta I_n^{\mathrm{U}} \tag{1.17}$$

$$\frac{\mathrm{d}R_n^{\mathrm{T}}}{\mathrm{d}t} = \zeta I_n^{\mathrm{T}} + \sum_{m=1}^{N}\left(w_{nm}R_m^{\mathrm{T}} - w_{mn}R_n^{\mathrm{T}}\right), \tag{1.18}$$

where

$$I_n = I_n^{\mathrm{U}}(t) + I_n^{\mathrm{T}}(t) \tag{1.19}$$

is the total infected population - treated and untreated - in n. Note that we have now eliminated the diffusion of the $\mathcal{R}^{\mathrm{U}}$ population in (1.17), as this state represents the individuals who have died from the disease, and are thus redacted from the traveling population.

The consumption rate ρ depends on the drug availability in n, being zero in the limit of extreme shortage and approaching unity if there is sufficient availability to meet the demand. Hence we denote the total number of available drug doses in n by $Q_n(t)$, and its per-capita availability by

$$\varphi_n(t) = \frac{Q_n(t)}{S_n^{\mathrm{U}}(t) + I_n^{\mathrm{U}}(t)}, \tag{1.20}$$

in which the denominator represents the time-dependent demand in n. We then define the rate ρ as

$$\rho(\varphi_n) = \begin{cases} \varphi_n & \text{for} \quad \varphi_n < 1 \\ 1 & \text{for} \quad \varphi_n \geq 1 \end{cases} = \min\{\varphi_n, 1\}, \tag{1.21}$$

which in Eqs. (1.13) - (1.16) ensures that drug doses are consumed as long as there is local demand, while avoiding excess consumption when $Q_n(t) > S_n^{\rm U}(t) + I_n^{\rm U}(t)$, in which case n has more available doses than required for its current untreated population.

To close the set of equations (1.13) - (1.18) we track the drug availability $Q_n(t)$ in each location n. Under centralized mitigation drug doses are manufactured and shipped from a single source node s at a rate $\mathcal{C}_s$ (doses/day), providing

$$\frac{{\rm d}Q_n}{{\rm d}t} = -\rho(S_n^{\rm U} + I_n^{\rm U}) + \theta(t - t_{\rm R})\delta_{ns}\mathcal{C}_s + \sum_{m=1}^{N}\Big(B_{nm}Q_m - B_{mn}Q_n\Big), \tag{1.22}$$

where $\delta_{ns} = 1$ if $n = s$ and $\delta_{ns} = 0$ otherwise. The delay function θ is the Heavyside step-function

$$\theta(x) = \begin{cases} 0 & \text{for} \quad x < 0 \\ 1 & \text{for} \quad x \geq 0 \end{cases}, \tag{1.23}$$

initiating drug production only at $t \geq t_{\rm R}$, incorporating the response time required before beginning drug distribution. The diffusion rates B_{nm} represent the fraction of drug-doses in m that ship daily to n; see Sec. 2.2.

Under decentralized mitigation drugs are synthesized locally, with each node characterized by its individual production rate $\mathcal{C}_n$, providing

$$\frac{{\rm d}Q_n}{{\rm d}t} = -\rho(S_n^{\rm U} + I_n^{\rm U}) + \theta(t - t_{\rm R})\mathcal{C}_n. \tag{1.24}$$

Hence, Eqs. (1.13) - (1.18) with ρ taken from (1.21) capture the spread of the epidemic and its competition with the distributed therapy. The distinction between centralized and decentralized mitigation is provided by the $Q_n(t)$ equations, taken from (1.22) or (1.24), respectively.

1.3 Normalized equations

Next we rewrite Eqs. (1.13) - (1.18) for the normalized populations $s_n^{\rm U}(t) = S_n^{\rm U}(t)/M_n$, $s_n^{\rm T}(t) = S_n^{\rm T}(t)/M_n$, $j_n^{\rm U}(t) = I_n^{\rm U}(t)/M_n$, $j_n^{\rm T}(t) = I_n^{\rm T}(t)/M_n$, $r_n^{\rm U}(t) = R_n^{\rm U}(t)/M_n$ and $r_n^{\rm T}(t) = R_n^{\rm T}(t)/M_n$, obtaining

$$\frac{{\rm d}s_n^{\rm U}}{{\rm d}t} = -\alpha s_n^{\rm U} j_n \sigma(j_n) - \gamma\rho s_n^{\rm U} + \sum_{m=1}^{N}\left(\frac{M_m}{M_n}w_{nm}s_m^{\rm U} - w_{mn}s_n^{\rm U}\right) \tag{1.25}$$

$$\frac{{\rm d}s_n^{\rm T}}{{\rm d}t} = -\alpha s_n^{\rm T} j_n \sigma(j_n) + \gamma\rho s_n^{\rm U} + \sum_{m=1}^{N}\left(\frac{M_m}{M_n}w_{nm}s_m^{\rm T} - w_{mn}s_n^{\rm T}\right) \tag{1.26}$$

$$\frac{{\rm d}j_n^{\rm U}}{{\rm d}t} = \alpha s_n^{\rm U} j_n \sigma(j_n) - \beta j_n^{\rm U} - \gamma\rho j_n^{\rm U} + \sum_{m=1}^{N}\left(\frac{M_m}{M_n}w_{nm}j_m^{\rm U} - w_{mn}j_n^{\rm U}\right) \tag{1.27}$$

$$\frac{\mathrm{d}j_n^{\mathrm{T}}}{\mathrm{d}t} = \alpha s_n^{\mathrm{T}} j_n \sigma(j_n) - \zeta j_n^{\mathrm{T}} + \gamma\rho j_n^{\mathrm{U}} + \sum_{m=1}^{N}\left(\frac{M_m}{M_n} w_{nm} j_m^{\mathrm{T}} - w_{mn} j_n^{\mathrm{T}}\right) \quad (1.28)$$

$$\frac{\mathrm{d}r_n^{\mathrm{U}}}{\mathrm{d}t} = \beta j_n^{\mathrm{U}} \quad (1.29)$$

$$\frac{\mathrm{d}r_n^{\mathrm{T}}}{\mathrm{d}t} = \zeta j_n^{\mathrm{T}} + \sum_{m=1}^{N}\left(\frac{M_m}{M_n} w_{nm} r_m^{\mathrm{T}} - w_{mn} r_n^{\mathrm{T}}\right), \quad (1.30)$$

where $j_n = j_n^{\mathrm{U}}(t) + j_n^{\mathrm{T}}(t)$.

Assuming a negligible fraction of one-directional trips, *i.e.* that immigration accounts for a marginal part of the overall international mobility, we can write $F_{nm} = F_{mn}$, stating that, on average, the number of passengers flying daily from m to n is the same as those flying from n to m (see Supplementary Section 2). This enables us, using (1.5) to write

$$F_{nm} = F_{mn} \Rightarrow w_{nm} M_m = w_{mn} M_n, \quad (1.31)$$

which allows us to further simplify Eqs. (1.25) - (1.30), bringing them to their final normalized form

$$\frac{\mathrm{d}s_n^{\mathrm{U}}}{\mathrm{d}t} = -\alpha s_n^{\mathrm{U}} j_n \sigma(j_n) - \rho s_n^{\mathrm{U}} + \sum_{m=1}^{N} A_{nm}\left(s_m^{\mathrm{U}} - s_n^{\mathrm{U}}\right) \quad (1.32)$$

$$\frac{\mathrm{d}s_n^{\mathrm{T}}}{\mathrm{d}t} = -\alpha s_n^{\mathrm{T}} j_n \sigma(j_n) + \rho s_n^{\mathrm{U}} + \sum_{m=1}^{N} A_{nm}\left(s_m^{\mathrm{T}} - s_n^{\mathrm{T}}\right) \quad (1.33)$$

$$\frac{\mathrm{d}j_n^{\mathrm{U}}}{\mathrm{d}t} = \alpha s_n^{\mathrm{U}} j_n \sigma(j_n) - \beta j_n^{\mathrm{U}} - \rho j_n^{\mathrm{U}} + \sum_{m=1}^{N} A_{nm}\left(j_m^{\mathrm{U}} - j_n^{\mathrm{U}}\right) \quad (1.34)$$

$$\frac{\mathrm{d}j_n^{\mathrm{T}}}{\mathrm{d}t} = \alpha s_n^{\mathrm{T}} j_n \sigma(j_n) - \zeta j_n^{\mathrm{T}} + \rho j_n^{\mathrm{U}} + \sum_{m=1}^{N} A_{nm}\left(j_m^{\mathrm{T}} - j_n^{\mathrm{T}}\right) \quad (1.35)$$

$$\frac{\mathrm{d}r_n^{\mathrm{U}}}{\mathrm{d}t} = \beta j_n^{\mathrm{U}} \quad (1.36)$$

$$\frac{\mathrm{d}r_n^{\mathrm{T}}}{\mathrm{d}t} = \zeta j_n^{\mathrm{T}} + \sum_{m=1}^{N} A_{nm}\left(r_m^{\mathrm{T}} - r_n^{\mathrm{T}}\right), \quad (1.37)$$

where

$$A_{nm} = \frac{M_m}{M_n} w_{nm} = w_{mn} \quad (1.38)$$

is the normalized human flux matrix.

Next we normalize Eqs. (1.22) and (1.24) by tracking the per-capita drug availability $q_n(t) = Q_n(t)/M_n$, which approaches unity when in n there is sufficient dosage for the

entire local population. For centralized mitigation we obtain (1.22)

$$\frac{\mathrm{d}q_n}{\mathrm{d}t} = -\rho(s_n^{\mathrm{U}} + j_n^{\mathrm{U}}) + \theta(t - t_{\mathrm{R}})\delta_{ns}\left(\frac{\mathcal{C}_s}{M_n}\right) + \sum_{m=1}^{N}\Big(Z_{nm}q_m - B_{mn}q_n\Big), \tag{1.39}$$

where ρ is taken from (1.21) and

$$\varphi_n(t) = \frac{Q_n(t)}{S_n^{\mathrm{U}}(t) + I_n^{\mathrm{U}}(t)} = \frac{q_n(t)}{s_n^{\mathrm{U}}(t) + j_n^{\mathrm{U}}(t)} \tag{1.40}$$

in (1.20) remains unchanged under the normalization. The matrix

$$Z_{nm} = \frac{M_m}{M_n}B_{nm} \tag{1.41}$$

represents the incoming flux of therapeutics from m to n.

We define the normalized central production rate as

$$C_s = \frac{\mathcal{C}_s}{\sum_{m=1}^{N} M_m} \tag{1.42}$$

which approaches $C_s = 1$ when s has the capacity to produce and ship enough doses for the entire global population each day. Under common conditions we expect $C_s < 1$. Using this normalization Eq. (1.39) becomes

$$\frac{\mathrm{d}q_n}{\mathrm{d}t} = -\rho(s_n^{\mathrm{U}} + j_n^{\mathrm{U}}) + \theta(t - t_{\mathrm{R}})\delta_{ns}\left(\frac{\Omega}{M_s}\right)C_s + \sum_{m=1}^{N}\Big(Z_{nm}q_m - B_{mn}q_n\Big), \tag{1.43}$$

where

$$\Omega = \sum_{m=1}^{N} M_m \tag{1.44}$$

is the global population. Note that we used the δ-function in the second term to substitute M_n in the denominator of (1.39) by M_s in (1.43). Our normalization introduces the prefactor Ω/M_s, which quantifies the global population in *units* of s's local population. This factor arises because under centralized mitigation, to meet the global demand, the source s must export doses in quantities that are of order Ω/M_s greater than its local population. Therefore, a *normalized* capacity of $C_s = 1$ translates to a *per-capita* in s production of Ω/M_s doses per day. Such level of production, which does not scale with M_s, but rather with Ω is, in the majority of cases, significantly higher than practically attainable, hence we expect, in real scenarios, that $C_s < 1$.

Under decentralized mitigation, Eq. (1.24) provides

$$\frac{\mathrm{d}q_n}{\mathrm{d}t} = -\rho(s_n^{\mathrm{U}} + j_n^{\mathrm{U}}) + \theta(t - t_{\mathrm{R}})c_n, \tag{1.45}$$

where $c_n = \mathcal{C}_n/M_n$ is the per-capita local synthesis rate. Here $c_n = 1$ translates to a

production and local distribution capacity of M_n doses per day, namely n can provide therapy to its entire local population. As opposed to C_s, this normalization scales with the local, rather than global, population, hence $C_s = 1$ in the single source node s and $\langle c_n \rangle = 1$ over all $n = 1, \ldots, N$, both capture the case where the global demand can be met within one day - in the former in a centralized fashion and in the latter, decentralized across all N nodes.

1.4 The human flux matrix

To construct A_{nm} from data we denote the mobility rate by [2]

$$\mu = \frac{\sum_{m,n=1}^{N} F_{nm}}{\sum_{n=1}^{N} M_n}, \tag{1.46}$$

in which the numerator quantifies the number of individuals flying per day and the denominator equals to the total population, therefore providing the daily fraction of individuals who seek air travel. This allows us to estimate a local population M_n by

$$M_n = \frac{1}{\mu} \sum_{k=1}^{N} F_{kn}, \tag{1.47}$$

assuming that all people departing from n, *i.e.* $\sum_{k=1}^{N} F_{kn}$, represent a μ fraction of n's total population, M_n. We can now write

$$w_{nm} = \frac{F_{nm}}{M_m} = \mu \frac{F_{nm}}{\sum_{k=1}^{N} F_{km}} \tag{1.48}$$

and

$$\frac{M_m}{M_n} = \frac{\sum_{k=1}^{N} F_{km}}{\sum_{k=1}^{N} F_{kn}}, \tag{1.49}$$

providing

$$A_{nm} = \mu \frac{F_{nm}}{\sum_{k=1}^{N} F_{kn}}, \tag{1.50}$$

which allows us to extract A_{nm} directly from mobility data (F_{nm}, μ).

A similar derivation, using Eq. (1.47), helps us express the ratio Ω / M_s in (1.43) as

$$\frac{\Omega}{M_s} = \frac{\sum_{m,n=1}^{N} F_{nm}}{\sum_{n=1}^{N} F_{ns}} \equiv \kappa_s. \tag{1.51}$$

The parameter κ_s captures s's fraction of the global population. For a typical source node s, it scales as

$$\langle \kappa_s \rangle \sim N, \tag{1.52}$$

the number of nodes in the air-traffic network.

Summary

Normalized equations for epidemic spreading with treatment:

$$\frac{\mathrm{d}s_n^{\mathrm{U}}}{\mathrm{d}t} = -\alpha s_n^{\mathrm{U}} j_n \sigma(j_n) - \gamma\rho(\varphi_n) s_n^{\mathrm{U}} + \sum_{m=1}^{N} A_{nm}\left(s_m^{\mathrm{U}} - s_n^{\mathrm{U}}\right) \tag{1.53}$$

$$\frac{\mathrm{d}s_n^{\mathrm{T}}}{\mathrm{d}t} = -\alpha s_n^{\mathrm{T}} j_n \sigma(j_n) + \gamma\rho(\varphi_n) s_n^{\mathrm{U}} + \sum_{m=1}^{N} A_{nm}\left(s_m^{\mathrm{T}} - s_n^{\mathrm{T}}\right) \tag{1.54}$$

$$\frac{\mathrm{d}j_n^{\mathrm{U}}}{\mathrm{d}t} = \alpha s_n^{\mathrm{U}} j_n \sigma(j_n) - \beta j_n^{\mathrm{U}} - \gamma\rho(\varphi_n) j_n^{\mathrm{U}} + \sum_{m=1}^{N} A_{nm}\left(j_m^{\mathrm{U}} - j_n^{\mathrm{U}}\right) \tag{1.55}$$

$$\frac{\mathrm{d}j_n^{\mathrm{T}}}{\mathrm{d}t} = \alpha s_n^{\mathrm{T}} j_n \sigma(j_n) - \zeta j_n^{\mathrm{T}} + \gamma\rho(\varphi_n) j_n^{\mathrm{U}} + \sum_{m=1}^{N} A_{nm}\left(j_m^{\mathrm{T}} - j_n^{\mathrm{T}}\right) \tag{1.56}$$

$$\frac{\mathrm{d}r_n^{\mathrm{U}}}{\mathrm{d}t} = \beta j_n^{\mathrm{U}} \tag{1.57}$$

$$\frac{\mathrm{d}r_n^{\mathrm{T}}}{\mathrm{d}t} = \zeta j_n^{\mathrm{T}} + \sum_{m=1}^{N} A_{nm}\left(r_m^{\mathrm{T}} - r_n^{\mathrm{T}}\right) \tag{1.58}$$

Extracting A_{nm} from (1.50).

Centralized mitigation:

$$\frac{\mathrm{d}q_n}{\mathrm{d}t} = -\rho(\varphi_n)(s_n^{\mathrm{U}} + j_n^{\mathrm{U}}) + \theta(t - t_{\mathrm{R}})\delta_{ns}\kappa_s C_s + \sum_{m=1}^{N}\left(Z_{nm} q_m - B_{mn} q_n\right) \tag{1.59}$$

Decentralized mitigation:

$$\frac{\mathrm{d}q_n}{\mathrm{d}t} = -\rho(\varphi_n)(s_n^{\mathrm{U}} + j_n^{\mathrm{U}}) + \theta(t - t_{\mathrm{R}}) c_n \tag{1.60}$$

Taking $\rho(\varphi_n)$ from (1.21) and κ_s from (1.51).

2 Implementation

2.1 Human mobility network and epidemic parameters

We used the Global Mobility Network dataset [8] to extract the empirical fluxes F_{nm}. The dataset lists the daily travel of $\sim 8.91 \times 10^6$ passengers per day over the course of 365 days, *i.e.* $i = 1, \dots, 365$, between $N = 1,292$ airports, linked through $38,377$ directional air-routes. The mean flux from m to n is thus captured by

$$F_{n \leftarrow m} = \frac{1}{365} \sum_{i=1}^{365} F_{n \leftarrow m}^{\{i\}} \tag{2.1}$$

where $F_{n \leftarrow m}^{\{i\}}$ is the number of passengers flying from m to n in day i. In our analysis we assume an almost symmetric flux matrix, as expressed in Eq. (1.31). Indeed we find that practically all links are bi-directional, and that in the vast majority of existing links we have $F_{n \leftarrow m} \approx F_{n \rightarrow m}$, a similar passenger flux in both directions. Therefore, to construct F_{nm} we used the symmetrized

$$F_{nm} = \frac{F_{n \leftarrow m} + F_{n \rightarrow m}}{2}, \tag{2.2}$$

a network comprising $19,614$ bi-directional links, that represents a symmetric approximation of the empirical fluxes. Evaluating the local populations M_n as in (1.47) we obtained A_{nm} via (1.50). Following Ref. [2], we used $\mu = 1.7 \times 10^{-3}$ day^{-1} for the mobility rate.

The disease parameters in (1.53) - (1.58) were set to $\alpha = 2$ day^{-1} and $\beta = 0.2$ day^{-1}, providing a reproduction rate of $\mathcal{R}_0 = \alpha/\beta = 10$, and hence ensuring a highly contagious pandemic, which, untreated, will be guaranteed to spread globally [3]. Other, less extreme scenarios, are examined in Sec. 4. The treated population recovery rate is set to $\zeta = 1$ day^{-1}, and the efficacy is set to $\gamma = 1$. For the penetration threshold in $\sigma(j_n)$ we used $\varepsilon = 10^{-6}$ and $h = 8$ in (1.6). The term $\Omega/M_s = \kappa_s$ in (1.59) was evaluated using (1.51). Finally, the response time $t_{\rm R}$ was set to be at the onset of the pandemic spread, evaluated as the time t when the infection levels have reached 10% of the projected peak infection (Fig. S1). For the parameters listed above, this provides $t_{\rm R} = 12$ days. As our source node we selected s=ITM (Itami International Airport, Osaka, Japan), testing the effect under other sources in Section 4.

The capacities C_s and c_n were set variably to test the performance of each method, centralized vs. decentralized with varying global/local capacities. In the case of decentralized mitigation the capacities are distributed, potentially taking different values in different locations n. Therefore, if *e.g.*, we set the mean capacities to $C_s = \langle c_n \rangle = C$, then $C_s = C$, but $c_n \sim \mathcal{N}(C, \sigma^2)$ is a random variable extracted from a normal distribution with mean C and standard deviation $\sigma = 0.1C$.

The above represents our default parameters, used, *e.g.*, to produce Fig. 1 of the main text; in Figs. 2 - 3 we consistently vary some of these parameters, such as $t_{\rm R}$ or γ as reported in the main text. Excluding the varied parameter in each panel, *e.g.*, $t_{\rm R}$ in Fig. 2a, the remaining parameters continue to be fixed at the above default values. The setting used to produce Fig. 4 of the main text are explicated in Sec. 3. In Sec. 4 we examine the behavior of our system under different disease parameter sets, varying the reproduction rate $\mathcal{R}_0 = \alpha/\beta$.

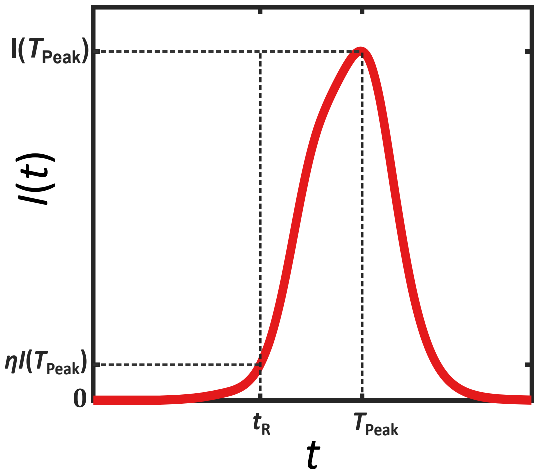

Figure S1: **Selecting the response time**. To select t_{R} we tracked the global level of infection, $I(t) = \sum_{n=1}^{N} I_n(t)$, vs. t, and set t_{R} to be the time where $I(t)$ has reached an η fraction of its peak value, namely $I(t_{\mathrm{R}}) = \eta I(T_{\mathrm{Peak}})$. Here we set $\eta = 0.1$.

In our simulations we track the epidemic spread using a 4th order Runge-Kutta stepper to iteratively solve Eqs. (1.53) - (1.60), with the relevant parameters as described above.

2.2 Drug distribution networks

The networks Z_{nm} and B_{mn} in (1.59) represent the fluxes of therapeutic doses to and from n respectively. These fluxes depend on the distribution strategy, which in turn impacts the efficiency of the drug dissemination. We consider three different constructions of these networks - two that build on the existing air-routes, and a third that achieves the highest efficiency by exerting extensive control over the international aviation network.

Diffusive distribution (Network 1). The results presented in the main text were obtained by setting

$$Z_{nm} = B_{mn} = \frac{F_{nm}}{\sum_{k=1}^{N} F_{km}}. \tag{2.3}$$

This represents diffusive distribution, in which the drug spreads along similar routes as the human travelers. The rationale is that in each node m the $q_m(t)$ available doses are split among each of m's outgoing destinations. The fraction of these doses to reach n is equal to the fraction of passengers departing from m whose destination is n, namely we calculate the flux from m to n (numerator) as a fraction of the total flux outgoing from m (denominator). This natural distribution scheme requires no intervention in the aviation networks, as the busy routes, mobilizing many human travelers, also naturally ship a larger fraction of commodities, in this case - drug doses. It also leads, on average, to a *fair* distribution, as airports with large incoming fluxes, which are typically highly exposed to the spreading pathogens, also receive increased fractions of the therapy. Under this construction, the networks $Z_{nm} = B_{mn}$ are symmetric, as they can both be expressed,

using Eq. (1.50), as

$$Z_{nm} = B_{mn} = \frac{1}{\mu} A_{nm}, \tag{2.4}$$

capturing an effective *travel* network in which 100% of *passengers*, in this case the available drug doses, travel each day (equivalent to setting $\mu = 1$ in (1.50), *i.e.* a mobility rate of 100%).

Directed distribution (Network 2). As an alternative to the above network we consider a direct flight network, in which the therapeutic is shipped in directed routes from s to all destinations. First we consider the binary air-route network G_{nm}, which satisfies

$$G_{nm} = \begin{cases} 1 & \text{if} \quad F_{nm} > 0 \\ 0 & \text{if} \quad F_{nm} = 0 \end{cases}, \tag{2.5}$$

capturing all existing air-routes, regardless of their volume of traffic. As the therapeutic is shipped from s to all destinations it relies on the existing routes G_{nm}, but avoids cyclic pathways that may bring it back closer to s. This resembles pre-planned routes, which, on the one hand, must rely on existing lines, but on the other hand seek the most efficient routing from source to destination, avoiding cyclic pathways. To construct this directed network, we first used the depth first search (DFS) algorithm [9] starting from the therapeutic source node s. At the first step one traverses along all existing G_{mn} links from s to its nearest neighbors, *i.e.* s's *first generation descendants*. One then continues to advance along the edges from each of these descendants, seeking additional *new* second generation descendants, then third generation and so on. In this process the G_{nm} links traversed in each step may be in one of four groups (Fig. S2a): *Tree links* - linking i-generation nodes to $i+1$-generation nodes; *Forward links* - linking i-generation nodes to $i+k$-generation nodes ($k > 1$); *Back links* - linking i-generation nodes to $i-k$-generation nodes ($k \geq 1$); *Cross links* - all remaining links, connecting separated branches of the spanning tree. Once we have covered the entire network we eliminate all Back links to obtain the s-dependent directed air-routes network $G_{nm}^{\{s\}}$, in which only the routes leading *away* from s remain. Applying this algorithm to the air-traffic network, starting from s=ITM we arrive at a directed $G_{nm}^{\{s\}}$ with $19,605$ directed links, out of the original G_{nm}, which comprised $38,377$ links, of which the vast majority were bi-directional, *i.e.* $G_{nm} = G_{mn}$.

To obtain the relative fluxes along the directed links of $G_{nm}^{\{s\}}$ we use the empirical fluxes of F_{nm} to construct the acyclic

$$F_{nm}^{\{s\}} = G_{nm}^{\{s\}} F_{nm}, \tag{2.6}$$

capturing the volume of (human) travel along each directed route. The available therapeutic at each node m is split among the existing outgoing routes, with flux proportional to $F_{nm}^{\{s\}}$. Indeed, the busiest routes, which carry the largest volume of human travelers are also the ones shipping the largest volume of commodities, in this case drug doses. This provides the outgoing fluxes from n to m as

$$B_{mn} = \frac{F_{mn}^{\{s\}}}{\sum_{k=1}^{N} F_{kn}^{\{s\}}}, \tag{2.7}$$

allowing us to construct Z_{nm} from (1.41), expressing M_n and M_m via (1.47).

Balanced distribution (Network 3). In the main text we have shown that the primary lacuna of commodity flow is that, relying on existing air-travel fluxes, it inevitably leads to an unbalanced distribution, in which few nodes receive the therapeutic in excess quantities, while others remain deprived. Hence we consider a balanced network, which calculates for each node, all the downstream destinations along its descending routes, and divides the therapeutics accordingly. This represents an improved, albeit not optimal, physical distribution scheme. Indeed, a fully optimal distribution scheme is extremely difficult to attain, and practically impossible to execute. In fact, even the currently proposed balanced scheme requires a level of control over the air-traffic network that is extremely unlikely to apply in realistic scenarios. Moreover, optimal schemes in general, and our balanced scheme in particular, require us to intervene in the *natural* flow of commodities, by enforcing external control over the volumes of shipments that travel between destinations, hence they potentially come at a tremendous economic price-tag. Still, we examine this *best case scenario*, assuming a fully controlled balanced distribution scheme, as a test case for centralized mitigation under a well-designed distribution network.

To construct the balanced network we repeated the process leading to Network 2, constructing G_{nm} from (2.5) and using DFS from the source s. This time, however, we only kept Tree links, leading to an optimized network, in which all target nodes $n = 1, \dots, N$ are linked to the source s via G_{nm}'s shortest paths. Hence we arrive at $G_{nm}^{\{s\}}$, in which there is a single directed path $\Pi(s \to n)$ from s to all target nodes n. This path includes a minimal number of flight transfers, as enabled by the existing routes in G_{nm}, hence representing an efficient distribution network, which adheres to the bounds of our current overall aviation capacity.

Each node n is then assigned a *branching index* K_n, quantifying the total population in all of the nodes that are downstream from n. To be specific, consider the path

$$\Pi(s \to i) = \left\{ s \xrightarrow{G_{ks}^{\{s\}}} k \xrightarrow{G_{lk}^{\{s\}}} l \dots m \xrightarrow{G_{im}^{\{s\}}} i \right\}, \tag{2.8}$$

providing the shortest sequence from s to the target node i. For some intermediate node m we define the group (Fig. S2b)

$$\mathcal{K}_m = \left\{ i \middle| m \in \Pi(s \to i) \right\}, \tag{2.9}$$

which consists of all nodes i whose shortest path from s traverses m, namely nodes that are *downstream* from m. Note that m itself is also included in $\mathcal{K}_m$. Additionally, if m is a *leaf* node, namely a node with no outgoing links then $\mathcal{K}_m = \{m\}$, including only the node m itself. The corresponding branching index is defined as

$$K_m = \sum_{i \in \mathcal{K}_m} M_i, \tag{2.10}$$

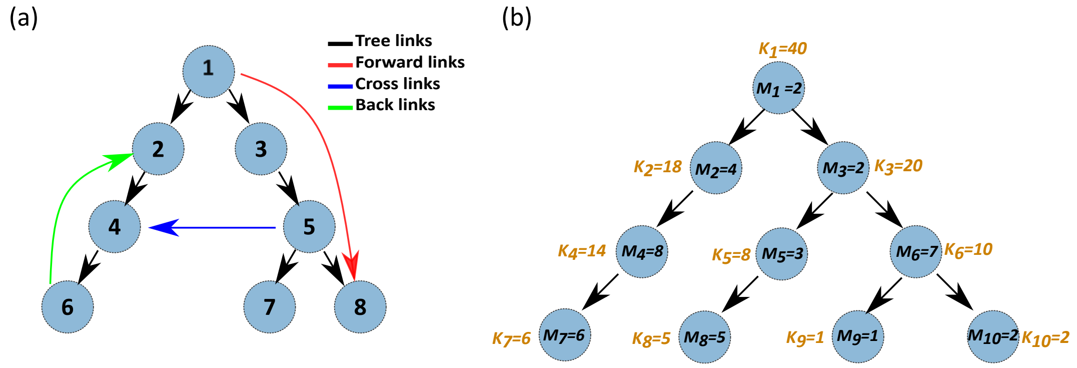

Figure S2: **Constructing the centralized distribution networks** B_{mn}. We constructed three distribution networks. (a) Diffusive (Network 1), comprising all links of the original air traffic network; Directed (Network 2), in which we eliminated Back links (green) to avoid cyclic pathways. Hence shipped doses avoid returning towards the source s (here represented by node number 1). Balanced (Network 3), in which we eliminated also Forward links (red) and Cross links (blue), providing a perfect tree network with a single directed route between s and all target nodes. (b) In Balanced (Network 3) each node n is assigned a branching index K_n quantifying the total population downstream from n, *i.e.* sum of M_i over all nodes i whose path from the source 1 traverses n. For example, the nodes downstream from 6 are $9, 10$ and 6 itself, hence $K_6 = M_6 + M_9 + M_{10} = 10$. To achieve a balanced spread of the therapeutic, each node must receive doses in quantities proportional to its branching index, to serve all its downstream target nodes. Therefore, in Network 3 the incoming link to n is proportional to K_n (Eq. (2.11)).

namely the total population in all nodes located downstream from m. Equation (2.10) captures the amount of doses that must be delivered from s to m if the entire population is to be served. The flux from n to m is then designed to be proportional to K_m, namely

$$B_{mn} = \frac{G_{mn}^{\{s\}} K_m}{\sum_{k=1}^{N} G_{kn}^{\{s\}} K_k}, \tag{2.11}$$

ensuring that of the available doses $q_n(t)$ at n, the share that reaches m is balanced with m's projected downstream shipments, amounting to K_m. As before Z_{nm} is constructed from (1.41).

In Fig. S3a we present $\epsilon_{\rm Res}$ vs. C as obtained for Networks 1 - 3 (orange, yellow, brown). For comparison we also show the results of decentralized mitigation (green). As expected decentralized mitigation achieves consistently better results compared to all three centralized distribution schemes. Among the centralized schemes the balanced Network 3 provides the best results, as expected by its egalitarian design. Interestingly Notwork 1, incorporating un-directed diffusive distribution is superior to the directed Network 2. The reason it that in the absence of cycles, a large volume of the available therapeutic is accumulated at the final destination nodes (leafs), becoming effectively lost. The diffusive Network 1 allows the drugs to continue cycling until reaching a destination where they are needed, and hence consumed. The Gini index is presented in Fig. S3b, showing a similar trend among the three centralized distribution strategies.

3 Heterogeneity in network-based distribution

3.1 The rate matrix ξ_{sn}

Consider the spread of a therapeutic under centralized mitigation via Eq. (1.59), namely

$$\frac{\mathrm{d}q_n}{\mathrm{d}t} = -\rho(\varphi_n)(s_n^{\mathrm{U}} + j_n^{\mathrm{U}}) + \theta(t - t_{\mathrm{R}})\delta_{ns}\kappa_s C_s + \sum_{m=1}^{N} \Big(Z_{nm} q_m - B_{mn} q_n \Big). \tag{3.1}$$

To track the supply distribution across all nodes let us ignore the local consumption, *i.e.* first term on r.h.s., and, for simplicity, set $t_{\mathrm{R}} = 0$, and the normalized capacity to $\kappa_s C_s = 1$. Using our diffusive spread of Eq. (2.3) we arrive at

$$\frac{\mathrm{d}q_s}{\mathrm{d}t} = 1 + \sum_{m=1}^{N} B_{ms}(q_m - q_s) \tag{3.2}$$

$$\frac{\mathrm{d}q_n}{\mathrm{d}t} = \sum_{m=1}^{N} B_{mn}(q_m - q_n) \qquad n \neq s, \tag{3.3}$$

in which one *unit* of the therapeutic is produced at s per unit time, then diffuses through the distribution network B_{mn} to reach all other nodes. To asses the rate of the incoming therapeutic at some destination n consider a path $\Pi(s \to n) = \{s \xrightarrow{B_{ms}} m \xrightarrow{B_{lm}} l \ldots k \xrightarrow{B_{nk}} n\}$, comprising a sequence of nodes and links along the drug distribution network from s to n. Each link B_{ij} in this pathway represents the fraction of doses present in j that will be routed to i, *i.e.* $B_{ij} = B_{i \leftarrow j}$. Hence a unit dosage exiting s that travels to n through $\Pi(s \to n)$ will be diluted to

$$\xi\big(\Pi(s \to n)\big) = \prod_{i,j \in \Pi(s \to n)} B_{ij}. \tag{3.4}$$

This represents the *rate* by which $\Pi(s \to n)$ delivers the therapeutic to n. In case there are multiple paths between s and n, the total rate ξ_{sn} can be approximated by the sum over all routes as

$$\xi_{sn} = \sum_{\Pi(s \to n)} \xi\big(\Pi(s \to n)\big). \tag{3.5}$$

For routes of length l this can be expressed as a matrix product $\xi_{sn}^{\{l\}} = [B^l]_{sn}$, *i.e.* the s, n entry of B^l. Therefore, the total rate of incoming therapeutics into a target node n is the sum over the contribution of all individual pathways, expressed by

$$\xi_{sn} = \left[\sum_{l=0}^{L_{\mathrm{Max}}} B^l \right]_{sn}, \tag{3.6}$$

as appears in Eq. (6) of the main text. Taking B_{nm} from the appropriate network (Net-

works 1 - 3) we obtain the rates ξ_{sn}, providing the amount of doses q_n reaching destination n per each unit shipped from the source s. The density function $P(\xi)$ captures the fraction of nodes whose rate is $\xi_{sn} \in (\xi, \xi + \mathrm{d}\xi)$. In Fig. S3c - e we present $P(\xi)$ as obtained from Networks 1 - 3, setting the source node to be $s =$ITM. We find that in our air-travel network the longest path has five legs, therefore we set $L_{\mathrm{Max}} = 5$ in (3.6).

A node n is considered *saved* ($\mathbf{S}_n = 1$) if, under treatment, its mortality rate is reduced to at least half of that observed in the absence of treatment, namely

$$\frac{r_n^{\mathrm{U}}(t \to \infty)}{r_n(t \to \infty)} \le \frac{1}{2}, \tag{3.7}$$

where $r_n^{\mathrm{U}}(t)$ is extracted from Eqs. (1.53) - (1.60) and $r_n(t)$ from the untreated (1.2) - (1.4). The probability of node n to be saved is strongly dependent on the availability of the therapeutic at n, and hence on its rate ξ_{sn}. To observe this we show in Fig. S3c - e the probability $P(\mathbf{S} \cap \xi)$ for a random node n to have $\xi_{sn} \in (\xi, \xi + \mathrm{d}\xi)$ *and* also have $\mathbf{S}_n = 1$. As predicted, we find that the uneven dilution strongly impacts the inequality in the $\mathbf{S}$-probability, where nodes with large ξ_{sn} have a much higher probability of being saved than those with small ξ_{sn}. The effect is less pronounced in Network 3, which is specifically designed to balance the inequality in B_{nm}.

3.2 Supply patterns in random networks

3.2.1 Network pathways

Consider a random symmetric binary network $G_{nm} = G_{mn}$ with an arbitrary degree-distribution $P(K)$, and randomly selected symmetric weights $W_{nm} = W_{mn}$. Combining these two matrices we construct a *model* transportation network $F_{nm} = G_{nm}W_{nm}$, resulting in a weighted network that is extracted from the configuration model ensemble [10]. We can now construct the consequent distribution network B_{mn}, whose weights capture the volume of commodity flow from n to m, via Eq. (2.3) as

$$B_{mn} = \frac{G_{nm}W_{nm}}{\sum_{k=1}^{N} G_{km}W_{km}}. \tag{3.8}$$

In (3.8) the weighted degrees $S_m = \sum_{n=1}^{N} B_{mn}$ are normalized to unity, as in Network 1 of Sec. 2.2, capturing the fact that all incoming commodities must also flow (or be consumed) out to other destinations, hence the total out-flux amounts to unity.

We define the un-weighted degree of m as

$$K_m = \sum_{n=1}^{N} G_{mn}, \tag{3.9}$$

counting the number of routes in/out of m, allowing us to express S_m as

$$S_m = K_m \left(\frac{1}{K_m} \sum_{n=1}^{N} B_{mn} \right) = K_m \left\langle B \right\rangle_m , \tag{3.10}$$

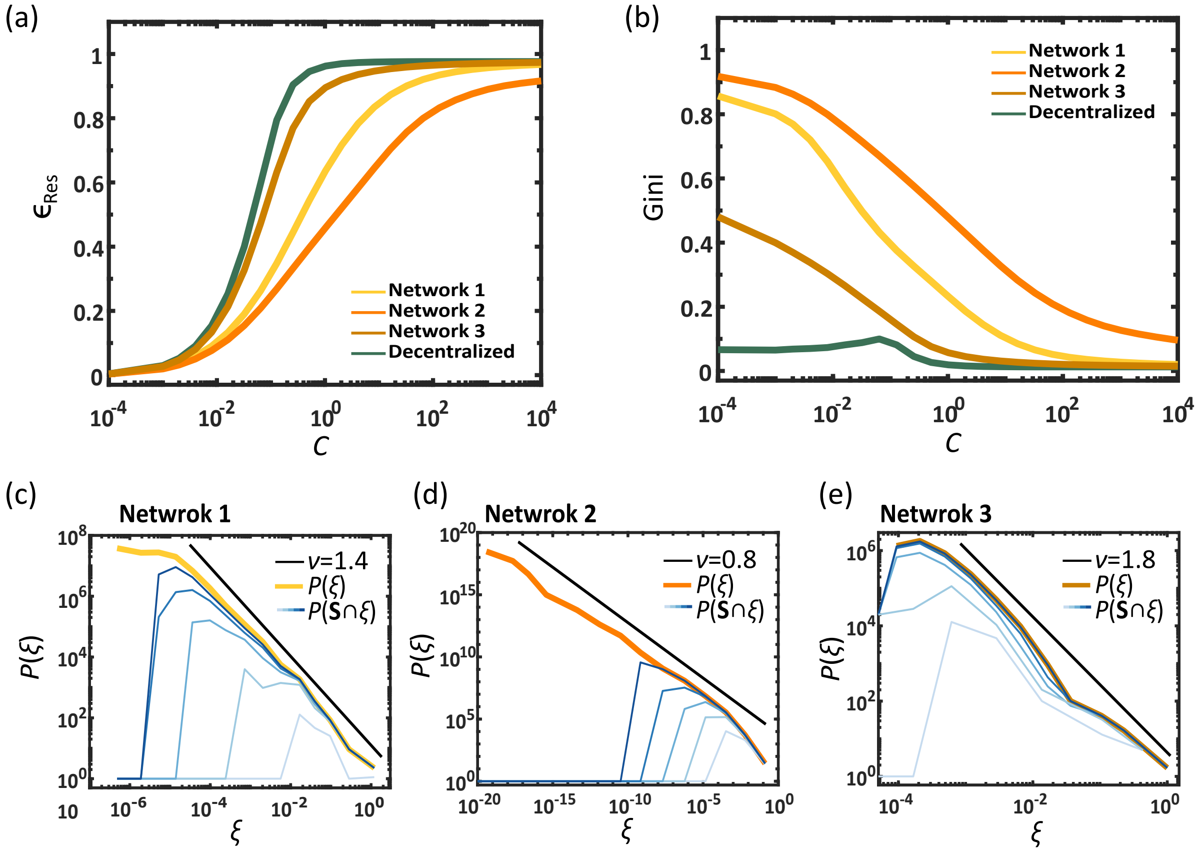

Figure S3: **The effect of the centralized distribution network** B_{mn}. We examined three constructions of B_{mn}: Network 1 (Diffusive), Network 2 (Directed) and Network 3 (Balanced). (a) - (b) ϵ_{Res} and the Gini coefficient vs. C for all networks. As expected Network 3 exhibits the highest efficiency and equality (brown) as it is specifically designed to generate an even spread of the therapeutic. Centralized mitigation (green), however, remains the optimal strategy. (c) - (e) $P(\xi)$ vs. ξ for all three networks. We find that ξ_{sn} is a determining factor of efficiency, with the high rate nodes having the highest probability of being saved. Shades of blue represent increasing capacities: Network 1 - $C = 0.01, 0.1, 0.3, 1.0, 3.0, 10$; Network 2 - $C = 0.002, 0.02, 0.2, 2.0, 20, 2000$; Network 3 - $C = 0.001, 0.01, 0.03, 0.06, 0.1, 0.3$. The effect of heterogeneity is most pronounced in Network 2, consistent with its lowest performance levels among the three networks. Note, that in Network 2 the power-law $P(\xi)$ is truncated for large ξ. As expected, heterogeneity is least pronounced in Network 3, which is designed for balanced distribution.

where $\langle B \rangle_m$ is the average weight of m's K_m outgoing links. Using the fact the $S_m = 1$ we can extract the average weight as

$$\langle B \rangle_m = \frac{1}{K_m}, \tag{3.11}$$

hence for a node with K_m outgoing routes, we have each route carrying, on average, $1/K_m$ of the available commodities for further shipping. Next we consider m's residual degree

$$K_{m,\odot} = \frac{1}{K_m} \sum_{n=1}^{N} G_{nm} K_n, \tag{3.12}$$

capturing the average un-weighted degree of m's nearest neighbors. Averaging over all nodes $(m = 1, \ldots, N)$ we obtain the network's mean residual degree

$$K_{\odot} = \frac{1}{N} \sum_{m=1}^{N} \left(\frac{1}{K_m} \sum_{n=1}^{N} G_{nm} K_n \right), \tag{3.13}$$

which expresses the average degree over all nearest neighbor nodes. This average neighbor degree is also commonly expressed as [10]

$$K_{\odot} = \frac{\langle K^2 \rangle}{\langle K \rangle}, \tag{3.14}$$

where $\langle K^n \rangle$ represents the n-th moment of $P(K)$. Equation (3.11) allows us to evaluate the average weight of a link associated with nearest neighbor nodes as

$$B_{\odot} = \frac{1}{K_{\odot}}. \tag{3.15}$$

The analysis above of the residual degree $K_{\odot}$ helps us characterize the pathways in G_{nm}, and hence, through (3.5), obtain the distribution of the rates ξ_{sn}. Indeed, the average node has $\langle K \rangle$ nearest neighbors, which, in turn, have, on average $K_{\odot}$ neighbors of their own. Therefore, a node has, on average, $\langle K \rangle K_{\odot}$ next neighbors and $\langle K \rangle K_{\odot}^2$ neighbors at distance $l = 3$, and so on. For a sufficiently large network, with $N \to \infty$, this predicts that the number of nodes at distance l from a randomly selected node grows as $N(l) = \langle K \rangle K_{\odot}^{l-1}$, an exponential growth that we approximate by [10]

$$N(l) \approx e^{\lambda l}, \tag{3.16}$$

where $\lambda = \ln K_{\odot}$. Equation (3.16) is valid as long as $l \lesssim \lambda^{-1} \ln N$, capturing the network's average path length $\langle l \rangle$, beyond which the *shells* $N(l)$ cease to grow exponentially. It follows from this equation that starting from an arbitrary source node s, the probability of a randomly selected node $n = 1, \ldots, N$ to be at distance $l_{sn} = l$ is

$$P(l) \sim e^{\lambda l}, \tag{3.17}$$

a valid approximation as long as $l \leq \langle l \rangle$.

3.2.2 Rate distribution $P(\xi)$

In a large random network, due to the local tree-like structure, there is often a single shortest path $\Pi(s \to n)$ between a source s and its target n, whose length is l_{sn}. The typical nodes along such path are, by definition, nearest neighbor nodes, and hence, on average their degree is $K_\odot$, and the l_{sn} weights linking between them are $B_\odot$. Consequently, the rate ξ_{sn} in (3.5) can be approximated by

$$\xi_{sn} = B_\odot^{l_{sn}} = K_\odot^{-l_{sn}}. \tag{3.18}$$

Therefore, on average, we have rates decaying exponentially with network distance l, as

$$\xi(l) = e^{-\lambda l}, \tag{3.19}$$

where, as above (Eqs. (3.16) and (3.17)) $\lambda = \ln K_\odot$. Equation (3.19) predicts that the average node at distance l from s receives the therapeutic at a rate proportional to $e^{-\lambda l}$, portraying an exponential dilution as the drugs propagate downstream from s.

We can now use (3.19) to obtain the density function $P(\xi)$, by transforming from the random variable ξ to the variable l, whose distribution is given by $P(l)$ in (3.17). Hence we write

$$P(\xi)\,\mathrm{d}\xi = P(l)\left|\frac{\mathrm{d}l}{\mathrm{d}\xi}\right|\mathrm{d}\xi. \tag{3.20}$$

Taking l from (3.19) we have $l = -\lambda^{-1}\ln\xi$ and hence $|\,\mathrm{d}l/\,\mathrm{d}\xi| \sim \xi^{-1}$. Using (3.17) and (3.19) we can also write $P(l) \sim \xi^{-1}$, hence, together we arrive at

$$P(\xi) \sim \xi^{-\nu}, \tag{3.21}$$

where $\nu = 2$, precisely the prediction of Eq. (7) in the main text.

This result is obtained under the configuration model ensemble, *i.e.* random network/weights. More generally, *e.g.*, in the presence of degree/weight correlations, the exponent ν can take different values. However the power-law form of (3.21) is expected under almost any network construction. Indeed, as long as the network exhibits an exponential growth of the form (3.17), and the downstream dilution exhibits an exponential decay of the form (3.19), we expect a scale-free $P(\xi)$. To observe this, consider, for example that the two exponential functions exhibit different growth/decay rates, *i.e.*

$$P(l) \sim e^{\lambda_1 l} \tag{3.22}$$

$$\xi(l) \sim e^{-\lambda_2 l}. \tag{3.23}$$

Under these conditions we continue to have $|\,\mathrm{d}l/\,\mathrm{d}\xi| \sim \xi^{-1}$, however $P(l)$ now follows $P(l) \sim \xi^{-\lambda_1/\lambda_2}$. Using these expressions in (3.20), we find that the scaling $P(\xi) \sim \xi^{-\nu}$ remains, only that now the scaling is

$$\nu = \frac{\lambda_1}{\lambda_2} + 1, \tag{3.24}$$

which expectedly provides $\nu = 2$ in case $\lambda_1 = \lambda_2$. Such generalized scaling ($\nu = 1.4$) was indeed observed in the empirical aviation network.

3.2.3 Critical capacity C_η

Consider the probability density $P(\xi)$. For a finite system of N nodes it has a minimal rate, $\xi_{\rm min}$, such that

$$\int_{\xi_{\rm min}}^{\infty} P(\xi)\,{\rm d}\xi = 1. \tag{3.25}$$

Denoting the normalization constant by Z, we express $P(\xi)$ as $P(\xi) \sim Z\xi^{-\nu}$, and use (3.25) to obtain

$$Z\int_{\xi_{\rm min}}^{\infty} \xi^{-\nu}\,{\rm d}\xi = 1 \Rightarrow Z = (\nu-1)\xi_{\rm min}^{\nu-1}. \tag{3.26}$$

The maximal rate for a system of N nodes can be obtained from $P(\xi \geq \xi_{\rm max}) = 1/N$, namely, the probability to exceed $\xi_{\rm max}$ is $1/N$, providing

$$Z\int_{\xi_{\rm max}}^{\infty} \xi^{-\nu}\,{\rm d}\xi = \frac{1}{N}, \tag{3.27}$$

and in turn

$$\xi_{\rm max}^{\nu-1} = \frac{NZ}{\nu-1}. \tag{3.28}$$

Taking Z from (3.26) we obtain

$$\left(\frac{\xi_{\rm max}}{\xi_{\rm min}}\right)^{\nu-1} = N. \tag{3.29}$$

At the same time, we can independently evaluate that $\xi_{\rm max} = \xi_{ss}$, namely the rate at the source itself, which is by definition equal to unity, and therefore

$$\xi_{\rm min} = N^{\frac{1}{1-\nu}}. \tag{3.30}$$

For example, under $\nu = 2$, we find that $\xi_{\rm min}$, which represents the rate at the majority of the nodes is approximately $1/N$, an extremely low rate in case $N \to \infty$.

Next we seek the critical capacity C_η to save an η-fraction of nodes. Consider a node n with rate ξ_{sn}. For each unit therapeutic produced at s, n receives a fraction ξ_{sn}, and hence under capacity C_s, the supply rate at n is of the order of $C_n = \xi_{sn}C_s$. The meaning is that the therapeutic availability at n, ignoring local consumption and just focusing on the supply from s, is, roughly, $q_n(t) \sim C_n t$. For n to be saved, *i.e.* $\mathbf{S}_n = 1$, we require $C_n \sim 1$, such that n is fully supplied ($q_n(t) = 1$) within a finite time. Therefore, the critical ξ_{sn} for saving n is approximately

$$\xi_{\rm Crit} = \frac{1}{C_s}. \tag{3.31}$$

To save an η fraction of all nodes we set C_s in (3.31) to $C_s = C_\eta$, namely a critical rate of $\xi_{\rm Crit} = 1/C_\eta$, and require that all nodes with $\xi_{sn} \ge \xi_{\rm Crit}$ are saved. Therefore, we write

$$Z \int_{\xi_{\rm Crit}}^{\infty} \xi^{-\nu}\, \mathrm{d}\xi = \eta, \tag{3.32}$$

setting the fraction of nodes whose rate exceeds the saving threshold $\xi_{\rm Crit}$ to η. We obtain

$$\frac{1}{\nu - 1} Z \xi_{\rm Crit}^{1-\nu} = \eta, \tag{3.33}$$

which, using (3.26), provides

$$\xi_{\rm Crit} = \eta^{\frac{1}{1-\nu}} \xi_{\rm min} \sim N^{\frac{1}{1-\nu}}, \tag{3.34}$$

where in the last step we used (3.30) to obtain $\xi_{\rm min}$'s scaling with N. Finally, we use (3.31) to convert $\xi_{\rm Crit}$ to the consequent critical capacity, yielding

$$C_\eta = \frac{1}{\xi_{\rm Crit}} \sim N^{\phi}, \tag{3.35}$$

where $\phi = 1/(\nu - 1)$, as appears in Eq. (8) of the main text.

3.2.4 Mean supply time $\langle T \rangle$

As explained above, under a given capacity C_s, node n, whose rate is ξ_{sn} receives a $C_n = C_s \xi_{sn}$ fraction of its demand per unit time. Therefore its supply time $T_{{\rm Supp},n}$ to fill up its demand (*i.e.* $q_n(t) = 1$), follows

$$T_{{\rm Supp},n} = \frac{1}{C_s \xi_{sn}} \sim \frac{1}{\xi_{sn}}. \tag{3.36}$$

This allows us to transform from $P(\xi)$ to $P(T)$, capturing the probability density to observe $T_{{\rm Supp},n} \in (T, T + \mathrm{d}T)$. We write

$$P(T)\,\mathrm{d}T = P(\xi) \left| \frac{\mathrm{d}\xi}{\mathrm{d}T} \right| \mathrm{d}T, \tag{3.37}$$

and, using $P(\xi) \sim \xi^{-\nu}$, $\xi \sim 1/T$, we arrive at $P(T) \sim T^{\nu-2}$. As before we seek the normalization factor Z_T via

$$Z_T \int_{T_{\rm min}}^{T_{\rm max}} T^{\nu-2}\, \mathrm{d}T = 1, \tag{3.38}$$

which provides

$$Z_T = \frac{\nu - 1}{T_{\rm max}^{\nu-1} - T_{\rm min}^{\nu-1}} \approx (\nu - 1) T_{\rm max}^{1-\nu}. \tag{3.39}$$

The final approximation, relying on $T_{\rm max} \gg T_{\rm min}$, is relevant as long as $\nu > 1$, a condition which is anyway necessary for $P(\xi)$ to be a valid (normalizable) density function. The mean supply time is given by

$$\langle T \rangle = Z_T \int_{T_{\min}}^{T_{\max}} T^{\nu-1} \, \mathrm{d}T \approx Z_T T_{\max}^{\nu}, \tag{3.40}$$

once again - neglecting the $T_{\min}$ limit. Taking Z_T from (3.39) we arrive at $\langle T \rangle \sim T_{\max}$, indicating that the mean supply time is governed by the latest supplied nodes. Indeed, given the scale-free nature of $P(\xi)$, the low ξ (and hence large T) nodes represent the majority of the destinations, and therefore they are the ones that determine the average supply times of the therapeutic. To estimate $T_{\max}$ we recall from (3.36) that it the inverse of $\xi_{\min}$. This allows us to use the scaling relationship of (3.30) to write

$$\langle T \rangle \sim \frac{1}{\xi_{\min}} \sim N^{\phi}, \tag{3.41}$$

with $\phi = 1/(\nu - 1)$, as before. Equation (3.41) is precisely the prediction of Eq. (10) in the main text.

4 Extended testing

4.1 The effect of the therapeutic distribution source

In centralized mitigation each outbreak is characterized by the disease origin at node o and by the therapeutic source from node s, where drugs are produced and shipped globally. In the main text we arbitrarily selected o to be the Bujumbura International Airport (BJM) in Burundi (Fig. S4a, red star), and s to be the Osaka International Airport (ITM) in Japan. In Fig. S4c - k we show $\epsilon_{\rm Res}$ and the Gini index vs. C as obtained from centralized vs. decentralized distribution for a selection of alternative sources. As expected, the specific source has little impact on the therapeutic efficiency, with decentralized consistently outperforming centralized. Ideally, one expects the best outcome to be observed if s and o are close to each other. Indeed, at the early stages of the outbreak the nodes in the vicinity of o are most severely impacted, and hence if these neighbors of o are efficiently treated, by, *e.g.*, setting s in close proximity, this may lead to a meaningful reduction in global mortality. This goal is best served if s is directly linked to o's neighborhood, in which case the local spread of the therapeutic is most efficient. To observe this we measured $\epsilon_{\rm Res}$ vs. ξ_{os}, providing an effective distance measure, along the paths from s to o. Setting $C = 0.2$, we find that, indeed, mortality is higher when ξ_{os} is small (Fig. S4b), however the effect is marginal.

4.2 The effect of disease parameters

In the main text we examine the effect of centralized vs. decentralized distribution under the extreme conditions of a highly fatal epidemic, in which $\mathcal{R}_0 = \alpha/\beta = 10$, conditions that, untreated, lead to an almost complete demise of the human population, *i.e.* $r_n(t \to \infty) \to 1$ for all n. In Fig. S5 we examine the results under varying disease parameters, freezing the recovery rate at $\beta = 0.2$ day^{-1}, and setting α such that $\mathcal{R}_0 = 2, 3, \ldots, 10$. As expected, we find that in all cases, decentralized mitigation consistently outperforms the centralized alternative. Note, that the parameter $\mathcal{R}_0$ not only impacts the level of contagion, but also affects the propagation times of the epidemic. This fact touches on the relevant selection of our response time $t_{\rm R}$, which must be adapted as $\mathcal{R}_0$ is changed. Indeed, setting *e.g.*, $t_{\rm R} = 12$ days, as we do for $\mathcal{R}_0 = 10$, would represent an unrealistically early response under $\mathcal{R}_0 = 2$, which, over the course of 12 days remains still in its early embryonic stage, likely not even detected as a potential threat. Therefore, to conduct a controlled examination of the impact of $\mathcal{R}_0$, we update our response time $t_{\rm R}$ as we change the disease parameters. We consistently select $t_{\rm R}$ to be the time t when the global infection level $I(t)$ has reached an η fraction of its peak value, setting $\eta = 0.1$ (Fig. S1).

5 Optimized physical distribution

5.1 Overview

To examine the potential efficiency of the therapuetic distribution under centralized mitigation we used the commodity flow framework [11] to design an optimal shipping strategy. We consider the air-traffic network as our underlying *flow network*, upon which the single *source* node s must ship the therapeutic in sufficient quantities, as given by the initial demands $d_n(0)$, to all *target* nodes $n = 1, \ldots, N$. Each link is characterized by a non-negative carrying capacity C_{nm}, capturing the maximal volume of the therapeutic that can be transferred from m to n through that link per unit time (day). Our goal is to meet the demand of all nodes as efficiently as possible, *i.e.* in minimum time, within the shipping constraints provided by C_{nm}.

An important consideration that arises from our earlier discussion is that distribution inequality can severely impact the efficiency of a therapeutic in battling the epidemic. Therefore we wish to distribute the therapeutic to *all* destinations in *parallel*, avoiding *serial* strategies that first fill up selected destinations, then continue shipping to others. To ensure such strategies are avoided we implemented a *day-by-day* optimization strategy, in which we maximize the commodity flow in each day $t = 1, \ldots, T$. Hence, at each day t we fill up as much of the current demand of *all* nodes as possible within that single day. We then update the remaining demand $d_n(t) \to d_n(t+1)$ and reiterate the process at day $t+1$, until all demands are satisfied at $t = T_{\max} = \max_{n=1}^{N}(T_{{\rm Supp},n})$.

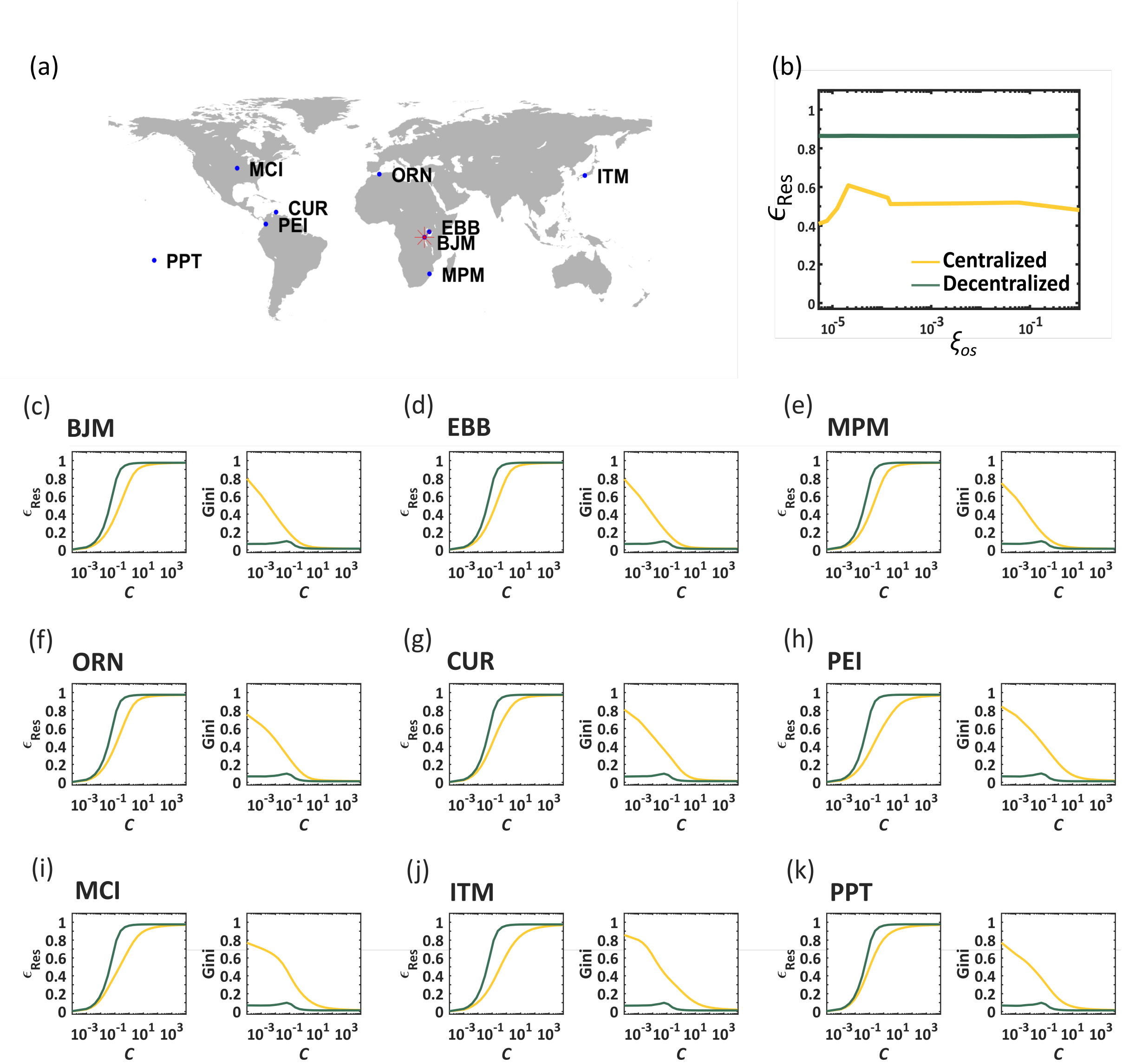

Figure S4: **The effect of the therapeutic distribution source** s. (a) Simulating an outbreak at o =BJM (red star) we selected 9 different sources s for the therapeutic distribution (blue dots). (b) The efficiency ϵ_{Res} vs. the rate ξ_{os} between the outbreak and the therapeutic source nodes ($C = 0.2$). (c) - (k) For each of the 9 sources we tested ϵ_{Res} and the Gini coefficient under centralized mitigation (yellow) and compared to decentralized mitigation (green).

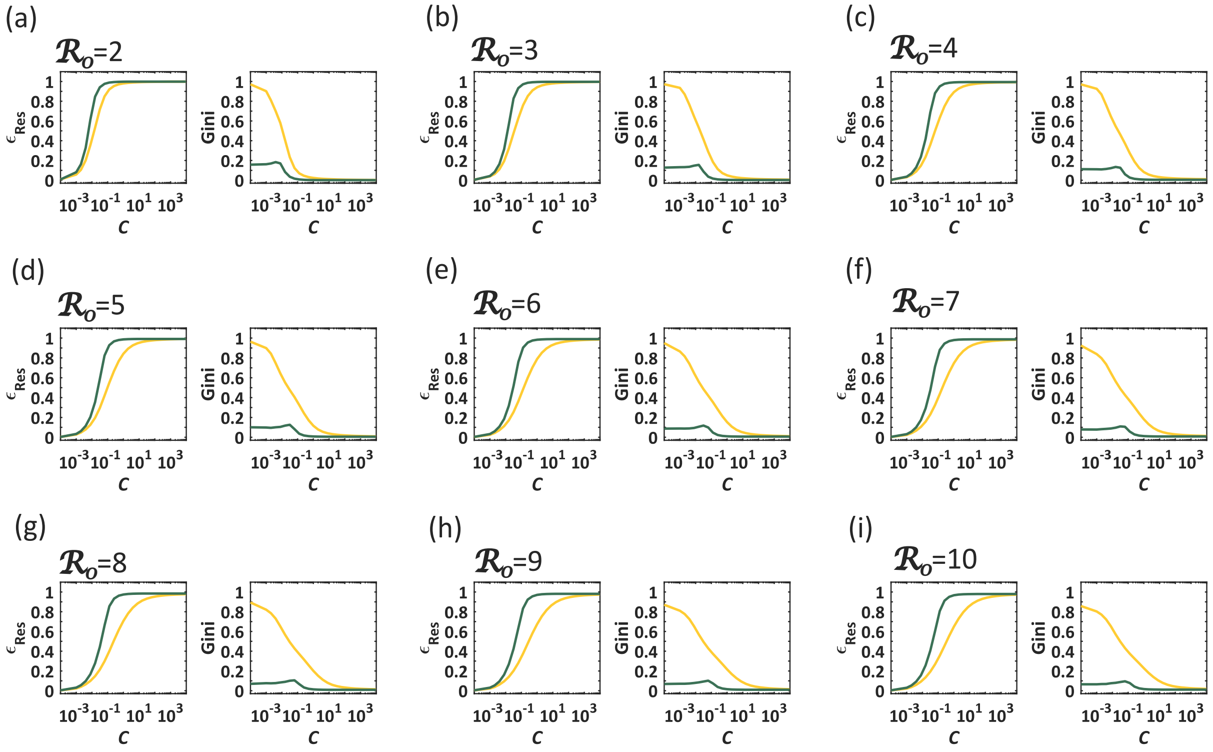

Figure S5: **The effect of disease parameters**. We tested decentralized (green) vs. centralized (yellow) mitigation for a range of disease parameters $\mathcal{R}_0 = 2, \ldots, 10$, finding that under all conditions decentralized outperforms centralized mitigation.

5.2 Maximum flow optimization

At $t = 0$ we select a source node s, and set the initial demands of all other nodes to $d_n(0) = M_n$, *i.e.* each node's demand is equal to its population size. In each day the therapeutic is distributed from s, aiming for *maximum flow* [12–14], namely seeking to maximize the number of doses exiting s and spreading to all other nodes. Denoting the volume of doses shipped from m to n on day t by $\mathcal{F}_{nm}(t)$, this optimization scheme translates to

$$\textbf{Maximize} \;\; \mathcal{F}_s(t) = \sum_{m=1}^{N} \big(\mathcal{F}_{ms}(t) - \mathcal{F}_{sm}(t)\big). \tag{5.1}$$

Using this notation the net supply of doses *entering* a target node n throughout day t is $-\mathcal{F}_n(t) = \sum_{m=1}^{N} [\mathcal{F}_{nm}(t) - \mathcal{F}_{mn}(t)]$, and hence at the end of each day the demand is reduced by precisely that amount (note that the minus sign in $-\mathcal{F}_n(t)$ represents *incoming* flux to n, a counter-intuitive notation, which is motivated by the fact that $\mathcal{F}_s(t)$ - positive flux - represents drugs *exiting* the source). Consequently, the n-demand at day t is given by

$$d_n(t) = M_n + \sum_{\tau=0}^{t-1} \mathcal{F}_n(\tau), \tag{5.2}$$

starting at $d_n(0) = M_n$, and reducing the demand due to the cumulative therapeutic influx as time advances. The maximization of (5.1) is subject to two constraints that must be satisfied at all t:

$$0 \le \mathcal{F}_{nm}(t) \le C_{nm} \qquad \text{For all } n, m = 1, \ldots, N \tag{5.3}$$

$$0 \le -\mathcal{F}_n(t) \le d_n(t) \qquad \text{For all } n = 1, \ldots, N, n \neq s. \tag{5.4}$$

The first constraint (5.3) ensures that all daily shipments from m to n do not exceed the carrying capacity C_{nm}; the second constraint (5.4), with $d_n(t)$ taken from (5.2), restricts nodes from accumulating therapeutics in excess of their remaining demand, therefore bounding the total incoming doses at day t, *i.e.* $-\mathcal{F}_n(t)$, by the current demand $d_n(t)$.

To solve the maximum flow optimization problem of (5.1) - (5.4) we used an iterative approach (Fig. 4a, main text): at each time step t we use linear optimization to maximize $\mathcal{F}_s(t)$ under the two constraints. This provides us with day t's optimized shipping strategy, detailing the daily fluxes $\mathcal{F}_{nm}(t)$ through all routes C_{nm}. With these fluxes we calculate the net influx into each node $-\mathcal{F}_n(t)$, and update the demands $d_n(t+1)$ in (5.2) accordingly. We then use these updated demands to obtain the shipping strategy for the next day $t+1$, repeating the process until all demands reach $d_n(T_{\text{Supp},n}) = 0$ at the final step $t = T_{\max}$. Note that in this process we disregard the flight times themselves, which, if accounted for, might incur additional delays. Instead we assume that the daily time unit accounts also for the lag of each route [15].

Implementation. To construct the carrying capacities C_{nm} we used the passenger fluxes F_{nm}, as obtained from the air travel data [8]. We assume that the daily volume of cargo shipped across the $m \to n$ route is proportional to the number of passengers F_{nm} for that route. This allows us to write

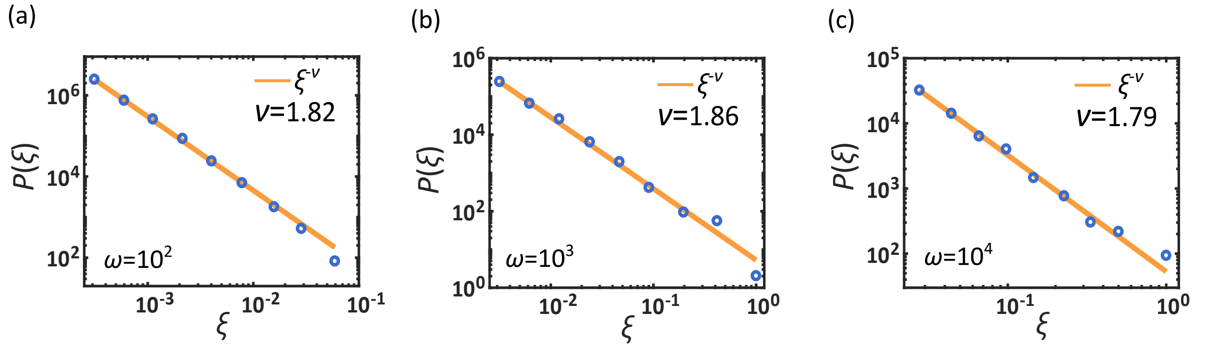

Figure S6: **The effect of the cargo carrying capacity**. We constructed C_{nm} in (5.5) using three different values for the passenger/cargo ratio ω, and used the maximum flow optimization of (5.1) - (5.4) to obtain the supply rate distribution $P(\xi)$. As expected the average rate increases as ω is increased, however the fat-tailed (power-law) nature of $P(\xi)$ remains unaffected, indicating that distribution inequality continues to govern the therapeutic dissemination.

$$C_{nm} = \omega F_{nm}, \tag{5.5}$$

where ω is the number of doses that can be shipped as cargo per traveling passenger. Our results are not sensitive to the specific value of ω, as any re-scaling of C_{nm} (capturing doses/day) is equivalent to an arbitrary re-scaling of the time units, having no bearing on the essential findings of Fig. 5 (main text). To select a realistic value, we assume each dose to have a gross weight of ~ 0.1 kg, which, taking the average passenger mass at ~ 100 kg, provides a ratio of $\omega \sim 10^3$. Therefore we selected ω in the range $10^2 - 10^4$ (Fig. S6), with the results in the main text obtained for $\omega = 10^3$. The initial demands were set to $d_n(0) = M_n$, extracting M_n from the data via (1.47).

References

[1] V. Colizza, A. Barrat, M. Barthélemy and A. Vespignani. The role of the airline transportation network in the prediction and predictability of global epidemics. *Proc. Natl. Acad. Sci. USA*, 103:2015, 2006.

[2] D. Brockmann and D. Helbing. The hidden geometry of complex, network-driven contagion phenomena. *Science*, 342:1337–1342, 2013.

[3] R. Pastor-Satorras, C. Castellano, P. Van Mieghem and A. Vespignani. Epidemic processes in complex networks. *Rev. Mod. Phys.*, 87:925–958, 2015.

[4] A. Barrat, M. Barthélemy and A. Vespignani. *Dynamical Processes on Complex Networks.* Cambridge University Press, Cambridge, 2008.

[5] P.S. Dodds and D.J. Watts. A generalized model of social and biological contagion. *Journal of Theoretical Biology*, 232:587–604, 2005.

[6] L. Hufnagel, D. Brockmann and T. Geisel. Forecast and control of epidemics in a globalized world. *Proc. Natl. Acad. Sci. USA*, 101:15124–9, 2004.

[7] L. Sattenspiel and K. Dietz. A structured epidemic model incorporating geographic-mobility among regions. *Mathematical Biosciences*, 128:71–91, 1995.

[8] OAG Ltd. 2018-06-11. *https://www.oag.com/.*

[9] T.H. Cormen, C.E. Leiserson, R.L. Rivest and C. Stein. *Introduction to Algorithms, Second Edition.* MIT Press and McGraw-Hill, Cambridge, MA., 2001.

[10] M.E.J. Newman. *Networks - an introduction.* Oxford University Press, New York, 2010.

[11] R.K. Ahuja, T.L. Magnanti and J.B. Orlin. *Network flows: theory, algorithms and applications.* Prentice Hall, 1993.

[12] L.R. Ford and D.R. Fulkerson. Maximal flow through a network. *Canadian Journal of Mathematics*, 8:399–404, 1956.

[13] L.R. Ford and D.R. Fulkerson. Constructing maximal dynamic flows from static flows. *Operations Research*, 6:419–433, 1958.

[14] A.V. Goldberg and R.E. Tarjan. Efficient maximum flow algorithms. *Communications of the ACM*, 57:82–89, 2014.

[15] L.K. Fleischer. Faster algorithms for the quickest transshipment problem. *SIAM Journal on Optimization*, 12:18–35, 2001.
